# Exploring Active Ingredients and Mechanisms of *Crataegi fructus* Extract in Alleviating MAFLD via the AMPK/PPAR Pathway by Multi-Omics

**DOI:** 10.3390/molecules31122118

**Published:** 2026-06-16

**Authors:** Xing Yan, Lulu Zheng, Yuexiang Xiao, Ya Xu, Qing Xu, Lihua Zeng, Siqi Hu, Deqing Ruan, Zhixin Wang

**Affiliations:** 1 Jiangxi Province Key Laboratory of Sustainable Utilization of Traditional Chinese Medicine Resources & Jiangxi Province Key Laboratory of Traditional Chinese Medicine Pharmacology, Institute of Traditional Chinese Medicine Health Industry, China Academy of Chinese Medical Sciences, Nanchang 330115, China; yanxing@itcmhi.ac.cn (X.Y.); zhenglulu@itcmhi.ac.cn (L.Z.); xuya@itcmhi.ac.cn (Y.X.); xuqing20000402@163.com (Q.X.); zenglihua@itcmhi.ac.cn (L.Z.); hsqjks@163.com (S.H.); 2 Jiangxi Health Industry Institute of Traditional Chinese Medicine, Nanchang 330115, China; 3 School of Pharmacy, Jiangxi University of Chinese Medicine, Nanchang 330004, China; xiaoyuexiang888@163.com; 4 Yunnan Key Laboratory of Southern Medicine Utilization, Yunnan University of Chinese Medicine, Kunming 650500, China

**Keywords:** metabolic dysfunction-associated fatty liver disease (MAFLD), lipid metabolism, *Crataegi fructus*, multi-omics, active ingredients

## Abstract

The fruit of *Crataegi fructus* (CF) is a traditional “medicine food” herb widely used for its lipid-lowering properties, but its active ingredients and mechanisms against metabolic dysfunction-associated fatty liver disease (MAFLD) remain poorly understood. This study employed an integrated multi-omics approach, combining serum metabolomics, liver transcriptomics, weighted gene co-expression network analysis (WGCNA), network pharmacology, and molecular docking, to systematically investigate the effects of CF extract (CFE) in a high-fat diet (HFD)-induced mouse model of MAFLD. Our analysis revealed that CFE treatment significantly reduced body weight gain (*p* < 0.01), improved glucose tolerance and insulin sensitivity (*p* < 0.01), and alleviated hepatic steatosis, as evidenced by reduced lipid accumulation and decreased NAS scores (*p* < 0.001). Metabolomics analysis showed that CFE reversed HFD-induced disturbances in serum fatty acids, glycerophospholipids, and bile acid metabolites. Transcriptomics further revealed that the AMPK and PPAR signalling pathways were critically involved in the regulation of lipid metabolism by which CFE alleviated MAFLD. Consistently, CFE treatment resulted in significant upregulation of AMPK and PPARα expression (*p* < 0.001) and downregulation of CD36 and DPP4 (*p* < 0.001), as confirmed by Western blotting and qPCR. Furthermore, integration of WGCNA and network pharmacology pinpointed chlorogenic acid (CA), ursolic acid (UA), and oleanolic acid (OA) as the primary bioactive components, and their lipid-lowering effects were validated in FFA-treated THLE-2 cells. In conclusion, this study offers preliminary insights into the lipid-lowering mechanisms of CFE via regulation of the AMPK/PPARα/CD36/DPP4 signalling pathway and support its further development as a functional food ingredient for MAFLD prevention.

## 1. Introduction

Metabolic dysfunction-associated fatty liver disease (MAFLD), also recently termed metabolic dysfunction-associated steatotic liver disease (MASLD) following international consensus recommendations, is characterised by excessive hepatic lipid accumulation and metabolic dysregulation [1]. Numerous studies have confirmed that MAFLD frequently coexists with obesity, type 2 diabetes mellitus (T2DM), and metabolic syndrome. This condition not only increases the risk of cardiocerebrovascular complications in patients with T2DM but also predisposes individuals to severe liver diseases, including fibrosis, cirrhosis, and even hepatocellular carcinoma (HCC) [2]. MAFLD has become one of the most common chronic liver diseases in clinical practice, affecting up to 38% of the global adult population [3]. The incidence of MAFLD-related HCC is rising at an annual rate of 9%, making it the third leading cause of HCC. Therefore, early prevention and treatment of MAFLD are of significant clinical and public health importance [4]. To date, however, only one therapeutic agent, resmetirom, has been approved by the FDA for the treatment of non-cirrhotic non-alcoholic steatohepatitis (NASH). Although semaglutide has shown promise in clinical trials for MAFLD, it has not yet received FDA approval specifically for this indication. Moreover, current clinical strategies mainly focus on managing advanced complications and often fail to reverse disease progression. Thus, there is an urgent need to identify novel, reliable, and safe alternative therapies for MAFLD.

*Crataegi fructus* (CF), the ripe fruit of the genus Crataegus in the Rosaceae family, is recognised as a “superfruit” [5], and has been used worldwide as both food and medicine [6]. Its medicinal use was first recorded in the Chinese Tang Dynasty pharmacopoeia Xin Xiu Ben Cao, and later mentioned by the Ancient Greek physician Pedanius Dioscorides in De Materia Medica (ca. 60–70 AD) for cardiovascular benefits. Today, several Crataegus species are officially included in the pharmacopoeias of China, Germany, and France [7]. In China, CF refers to *Crataegus pinnatifida* Bge. var. *major* N. E. Br. or *Crataegus pinnatifida* Bge., and is widely used as a natural herb. It contains diverse bioactive phytochemicals, including polyphenols (e.g., chlorogenic acid, procyanidin B2, epicatechin), flavonoids (e.g., quercetin, rutin), pentacyclic triterpenoids (e.g., ursolic acid, crataegolic acid, oleanolic acid), and glycosides. Owing to its hypoglycaemic, hypolipidaemic, antioxidant, anti-atherosclerotic, and antibacterial properties, CF has been extensively employed in the treatment of various diseases [8,9]. Previous studies have shown that CF polysaccharides significantly ameliorate hepatic steatosis, inflammatory infiltration, and liver injury in high-fat diet (HFD)-induced mice [10,11]. Hawthorn leaf flavonoids alleviate non-alcoholic fatty liver disease by enhancing the adiponectin/AMPK pathway [12]. Polyphenols, a class of micronutrients abundant in plant-based foods, improve MAFLD via gut microbiota modulation, as well as anti-inflammatory and antioxidant effects [13]. Additionally, CF extract and its flavonoids (e.g., rutin and quercetin) have been demonstrated to exert therapeutic effects against MAFLD by regulating purine metabolism and inhibiting the ERK/MAPK signalling pathway [9]. Nevertheless, the precise mechanisms and active components responsible for these therapeutic benefits remain insufficiently understood. Furthermore, different extraction methods yield distinct chemical profiles of CF, which directly affect its efficacy. Therefore, it is essential to evaluate the lipid-lowering effects of CF under defined extraction conditions, identify its active ingredients, and further elucidate its mechanism of action in regulating lipid metabolism.

Multi-omics analysis provides a systematic and comprehensive perspective that overcomes the limitations of single-omics approaches. It enables the holistic characterisation of biological network changes in response to drug intervention, reveals multi-level and latent molecular associations and pathways, and facilitates in-depth elucidation of therapeutic mechanisms [14]. Metabolomics is a key systems biology technique that examines dynamic changes in endogenous small-molecule metabolites, offering a global view of the metabolic landscape [15]. Transcriptomics investigates biological processes and gene expression regulation at the RNA level, providing a broad perspective on gene expression alterations [16]. Network pharmacology is an interdisciplinary field integrating bioinformatics, computational modeling, and molecular network data. It serves as an emerging data-mining tool for drug discovery, allowing systematic exploration of potential therapeutic mechanisms through the construction of component–target–pathway–disease networks. Weighted gene co-expression network analysis (WGCNA) is a bioinformatics method used to analyze gene expression data, identifying gene modules associated with specific diseases by examining co-expression patterns, thereby elucidating regulatory relationships and biological functions among genes [17]. Integrative multi-omics analysis is essential for elucidating the complex mechanisms of diseases [18]. For example, Zheng et al. employed a multi-omics approach to reveal that bound polyphenols in whole grain dietary fibre exert anti-obesity effects by increasing the abundance of beneficial bacteria such as *Akkermansia* and *Butyricicoccus*, and upregulating the expression levels of butyric acid and propionic acid [19]. Han et al. used a multi-omics strategy to discover that licochalcone A from licorice activates the PPAR signalling pathway and ameliorates glucolipid metabolic disorders in T2DM [20].

This study aimed to investigate the effects of CFE on MAFLD and to systematically explore its active components and underlying mechanisms using a multi-omics approach. To this end, we employed an integrated strategy combining metabolomics, transcriptomics, network pharmacology, WGCNA, and molecular biology techniques. We hypothesised that CFE alleviates MAFLD by modulating lipid metabolism pathways and the AMPK/PPAR signalling axis. To test this hypothesis, we first evaluated the anti-steatotic and hypolipidaemic effects of CFE in HFD-induced MAFLD mice, confirming its efficacy in ameliorating key features of the disease. Metabolomics analysis revealed that CFE rectified disturbances in serum fatty acids, glycerophospholipids, and bile acid metabolites. Next, transcriptomics indicated that the protective mechanism of CFE is closely associated with the regulation of lipid metabolism and the AMPK/PPAR signalling pathway. Consistently, molecular biology experiments confirmed that CFE significantly upregulated the expression of AMPK and PPARα, while downregulating DPP4 and CD36 in the liver of MAFLD mice. To further decode the substance basis of these effects, we employed WGCNA, network pharmacology, and molecular docking to predict potential active components, followed by in vitro experiments to validate their lipid-lowering activities. Collectively, this study provides a systems-level scientific basis for the use of CFE in MAFLD management and supports the development of this traditional medicine–food dual-use substance into modern precision functional foods and dietary intervention strategies.

## 2. Results

### 2.1. CFE Reduces Bodyweight Gain and Improves Glucose Metabolism in HFD-Fed MAFLD Mice

**Figure 1:**
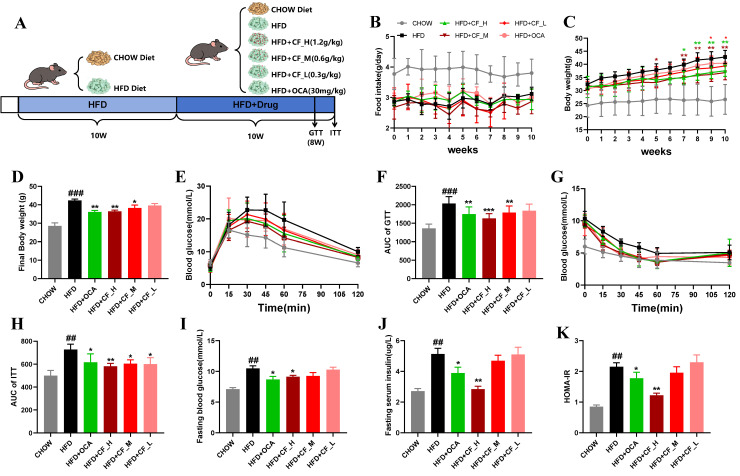
CFE suppresses body weight gain and enhances glucose metabolism in MAFLD mice. (**A**) Schematic representation of the experimental procedure. Mice were randomly allocated to six groups. Those fed a chow diet received oral gavage of normal chow. (**B**) Daily food consumption. (**C**) Dynamic changes in body weight across ten weeks. (**D**) Final body weight per group. (**E**) Glucose tolerance test (GTT) results from 0 to 120 min, and (**F**) corresponding area under the curve (AUC) following intraperitoneal administration of D-glucose (2 g/kg). (**G**) Insulin tolerance test (ITT) results from 0 to 120 min, and (**H**) AUC after intraperitoneal insulin injection (0.75 U/kg). (**I**–**K**) Fasting blood glucose (FBG), fasting serum insulin (FINS), and the homeostatic model assessment of insulin resistance (HOMA-IR). Data are expressed as mean ± SD (*n* = 9). Statistical significance was assessed using one-way ANOVA. ## *p* < 0.01, ### *p* < 0.001 vs. chow group; * *p* < 0.05, ** *p* < 0.01, *** *p* < 0.001 vs. HFD group.

To establish the MAFLD phenotype, mice were fed an HFD for 10 weeks, during which CFE was concurrently administered (Figure 1A). As expected, the HFD-fed group showed significant increases in final body weight, fasting blood glucose (FBG), and fasting serum insulin (FINS) compared with the CHOW group, whereas CFE intervention markedly attenuated these indicators in a dose-dependent manner. Notably, food intake remained stable across the three CFE dose groups, suggesting no appetite-suppressing effect (Figure 1B). Weekly body weight measurements revealed that CFE at 1.2 and 0.6 g/kg, as well as the positive control OCA at 30 mg/kg, reduced body weight in MAFLD mice relative to the HFD group (Figure 1C,D). To evaluate the effects of CFE on glucose metabolism, GTT and ITT were assessed. Compared with chow-fed controls, HFD-fed mice exhibited impaired glucose tolerance and insulin sensitivity, both of which were markedly improved by high-dose CFE intervention (Figure 1E–H). Consistently, HFD feeding led to substantial increases in FBG and FINS concentrations (Figure 1I,J), resulting in a high homeostatic model assessment of insulin resistance (HOMA-IR) index (Figure 1K). Overall, CFE effectively reversed these disturbances in glucose and insulin homeostasis.

### 2.2. CFE Attenuates Hepatic Lipid Accumulation and Improves Systemic Lipid in HFD-Fed MAFLD Mice

**Figure 2:**
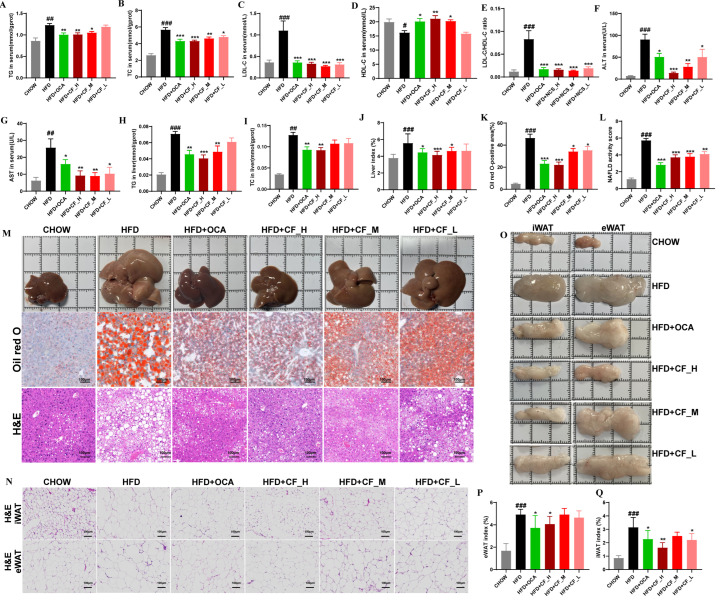
CFE alleviates hepatic fat accumulation and improves circulating lipid profiles in MAFLD mice. (**A**–**D**) Serum concentrations of TG, TC, LDL-C, and HDL-C. (**E**) LDL-C to HDL-C ratio. (**F**,**G**) Serum ALT and AST activities. (**H**,**I**) Hepatic contents of TG and TC. (**J**) Liver index (liver weight/body weight × 100%). (**K**) Quantification of Oil Red O staining area. (**L**) MAFLD activity score (NAS). (**M**) Representative images of gross liver morphology, H&E staining, and Oil Red O staining (original magnification, ×100). (**N**) H&E staining of iWAT and eWAT (×100). (**O**) Gross photographs of iWAT and eWAT. (**P**,**Q**) iWAT and eWAT indices (WAT weight/body weight × 100%). Data are expressed as mean ± SD (*n* = 9). Statistical significance was evaluated using one-way ANOVA. # *p* < 0.05, ## *p* < 0.01, ### *p* < 0.001 vs. chow group; * *p* < 0.05, ** *p* < 0.01, *** *p* < 0.001 vs. HFD group.

HFD feeding induced hepatic steatosis and lipid accumulation [21]. To comprehensively evaluate the effects of CFE on lipid metabolism, serum and liver biochemical parameters were measured after the 10-week intervention. As shown in Figure 2A–E, HFD-fed mice exhibited elevated serum TG, TC, and LDL-C concentrations, along with reduced HDL-C concentrations, all of which were effectively reversed by CFE intervention. Similarly, serum AST and ALT activities were higher in HFD-fed mice than in normal controls, and CFE treatment ameliorated these changes, indicating that hepatic lipid damage was alleviated to some extent (Figure 2F,G). Furthermore, CFE significantly reduced hepatic TG and TC concentrations in HFD-fed mice, suggesting alleviation of liver lipid accumulation (Figure 2H,I).

In addition, prolonged HFD feeding resulted in pronounced liver enlargement and an increased liver index compared with normal controls, whereas CFE treatment significantly reduced liver volume, weight, and index (Figure 2J,M). The NAS scores in the HFD group were above 5; in contrast, the CFE group maintained well-preserved hepatocyte architecture, with scant lipid droplets and only mild inflammation, resulting in NAS values substantially lower than those in the HFD group (Figure 2L). H&E staining showed normal hepatic morphology in the CHOW group, featuring a reddish-brown, smooth capsule with sharp edges and a soft, elastic consistency. By contrast, HFD-fed mice exhibited severe tissue injury, including disorganised hepatocytes, extensive macrovesicular steatosis accompanied by abundant lipid droplets, and moderate inflammatory infiltration, all of which were effectively reversed by CFE treatment (Figure 2M). Oil Red O staining further confirmed massive fat deposition in the livers of HFD-fed mice, while CFE treatment markedly reduced hepatic lipid content (Figure 2K,M). Moreover, mice in the CFE (1.2 g/kg) group exhibited significantly reduced eWAT and iWAT weights (Figure 2P,Q), along with a marked decrease in adipocyte size (Figure 2O). Consistently, H&E staining of eWAT and iWAT sections confirmed that CFE treatment minimised adipocyte hypertrophy (Figure 2N).

### 2.3. CFE Regulates Serum Metabolic Disturbances in HFD-Fed MAFLD Mice

**Figure 3:**
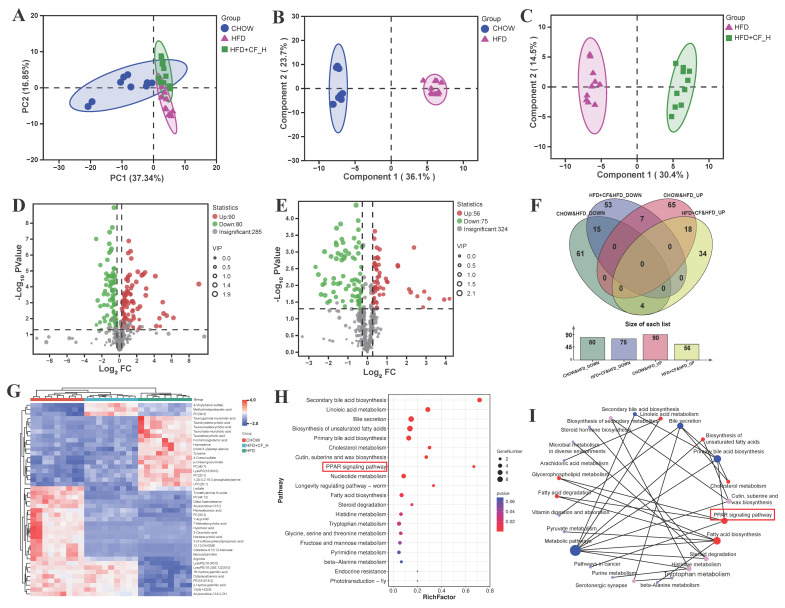
CFE regulates serum metabolic disturbances in HFD-fed MAFLD mice. (**A**) PCA score plot of the Chow, HFD, and HFD+CF_H groups. (**B**,**C**) OPLS-DA plot and 200-permutation test plot in metabolomics analysis, Chow vs. HFD (R^2^Y = 0.864, Q^2^ = 0.834) (**B**) and HFD vs. HFD+CF_H (R^2^Y = 0.904, Q^2^ = 0.859) (**C**). (**D**) Volcano plot of differentially accumulated metabolites (DAMs) (upregulated and downregulated) between Chow and HFD groups. (**E**) Volcano plot of DAMs between HFD and HFD+CF_H groups. (**F**) Venn diagram showing overlapping and unique differential metabolites between Chow vs. HFD and HFD vs. HFD+CF_H. (**G**) Heatmap of DAMs. (**H**) KEGG pathway enrichment analysis of the significant metabolites. (**I**) Association network of KEGG pathways.

Based on the above pharmacodynamic results, serum untargeted metabolic profiles were analysed across three groups: CHOW, HFD, and HFD+CF_H. In ESI+ and ESI− modes, a total of 1418 and 1313 features were identified, respectively (Supplementary Figure S1). Principal component analysis (PCA) was performed to discern intrinsic sample grouping. As shown in the PCA score plot (Figure 3A), the three groups exhibited distinct separation, with the first two principal components (PC1 and PC2) accounting for 37.34% and 16.85% of the total variance, respectively, indicating good representativeness for classification. Notably, HFD samples shifted rightward relative to the CHOW group, whereas CF-treated samples tended to revert toward the normal profile, suggesting that CFE promotes restoration of normal serum metabolites. Orthogonal partial least squares discriminant analysis (OPLS-DA) further revealed clear separations between CHOW and HFD (Figure 3B) as well as between HFD and HFD+CF_H (Figure 3C). The predictive performance of the OPLS-DA models was evaluated using R^2^Y and Q^2^ parameters, both of which approached 1, indicating that the models were well-fitted and exhibited strong predictive capability. Additionally, a series of 200 permutation tests was performed to assess model robustness. Volcano plot analysis (VIP > 1.0, |log_2_FC| > 0.3, −log_10_ (*p*-value) > 1.3) identified 90 upregulated and 80 downregulated metabolites in CHOW vs. HFD (Figure 3D), and 56 upregulated and 75 downregulated metabolites in HFD+CF_H vs. HFD (Figure 3E). A Venn diagram illustrated the number of varying metabolites among groups (Figure 3F).

Trend analysis identified 44 differentially accumulated metabolites (DAMs) (Figure 3G), and detailed information on these DAMs is provided in Supplementary Data S1. This set of metabolites encompasses several biologically significant classes, including fatty acids, glycerophospholipids, bile acids, and amino acids. Their relative abundances across the different experimental groups are depicted in the heatmap (Figure 3G). Notably, the DAMs identified in this analysis often exhibit similar and complementary biological functions, with analogous expression patterns observed between the CFE-treated and CHOW groups. Subsequently, these DAMs were subjected to pathway enrichment analysis and functional annotation to identify the metabolic pathways associated with MAFLD. The top 20 KEGG enrichment pathways are shown in Figure 3H. The results indicated that the enriched metabolic pathways primarily include linoleic acid metabolism, biosynthesis of unsaturated fatty acids, bile secretion, and the PPAR signalling pathway, among others. Further pathway network analysis revealed that these DAM-enriched pathways fell into three categories: lipid metabolism (e.g., linoleic acid metabolism, PPAR signalling), central carbon metabolism (e.g., glycolysis/gluconeogenesis), and amino acid metabolism (e.g., tryptophan metabolism) (Figure 3I).

### 2.4. RNA-Seq Analysis of Gene Expression Changes in HFD-Fed Mice Treated with CFE

**Figure 4:**
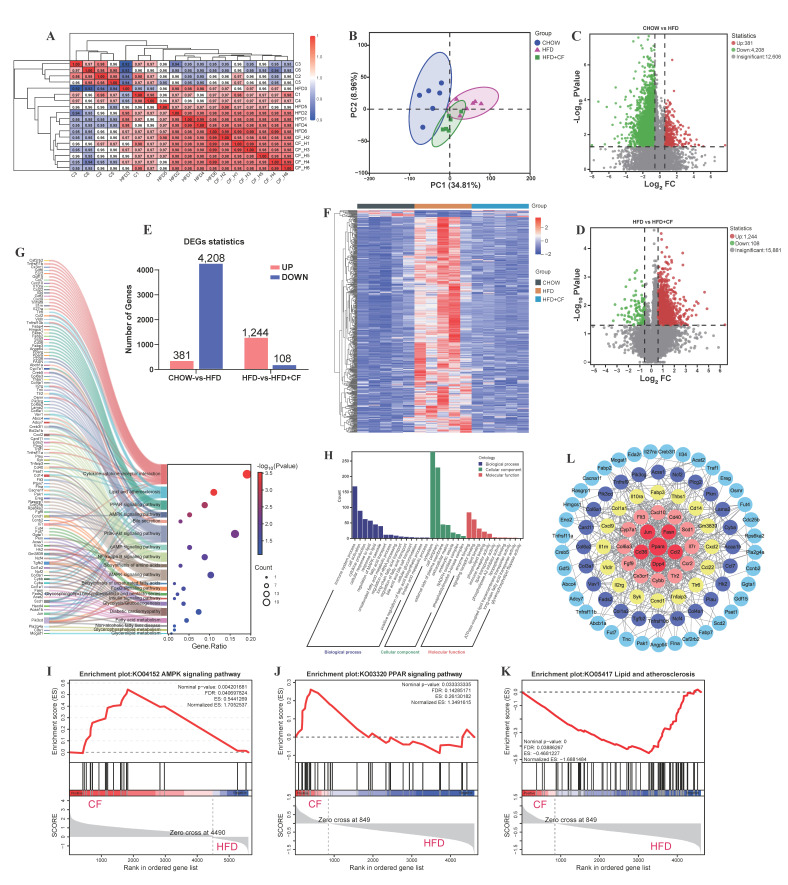
RNA-Seq analysis of transcriptomic changes in MAFLD mice following CFE treatment. (**A**) Inter-sample correlation analysis. (**B**) PCA of the Chow, HFD, and HFD+CF_H groups. (**C**) Volcano plot depicting differentially expressed genes (DEGs) between the Chow and HFD groups (indicating up- and downregulated genes). (**D**) Volcano plot of DEGs between the HFD and HFD+CF_H groups. (**E**) Quantitative summary of up- and downregulated DEGs. (**F**) Heatmap showing the expression profiles of DEGs. (**G**) Sankey diagram representing KEGG pathway enrichment analysis. (**H**) Gene Ontology (GO) enrichment results. (**I**–**K**) Gene set enrichment analysis (GSEA) plots. (**L**) Protein–protein interaction (PPI) network of the validated key DEGs.

To investigate the potential molecular pathways through which CFE exerts its effects on MAFLD, transcriptomic analysis was performed on liver samples from CHOW, HFD, and HFD+CF_H groups. Sample correlation analysis revealed intra-group consistency with correlation coefficients > 0.9 (Figure 4A), and PCA showed clear segregation among the three groups, indicating that both model establishment and drug intervention influenced transcriptional profiles (Figure 4B). Notably, HFD samples shifted rightward relative to CHOW, whereas CFE-treated samples tended to revert toward the normal profile, suggesting that CFE promotes restoration of normal hepatic gene expression.

Differentially expressed genes (DEGs) were defined using criteria of −log_10_(*p*-value) > 1.3 and |log_2_ FC| > 0.5 (Figure 4C,D). A total of 4589 DEGs were identified when comparing the CHOW and HFD groups (381 upregulated, 4208 downregulated), while 1352 DEGs were found between the HFD+CF_H and HFD groups (1244 upregulated, 108 downregulated). Among these, 400 genes showing similar expression patterns between the CF_H-treated group and the CHOW group were selected (Figure 4E). Their expression profiles are displayed in a heatmap constructed from normalized Z-scores (Figure 4F); detailed information on the DEGs is available in Supplementary Data S2. The hierarchical heatmap demonstrated clear clustering both across the three experimental groups and between upregulated and downregulated genes. GO functional enrichment analysis (Figure 4H) indicated that, in comparison with the HFD group, the DEGs in the CFE group were mainly enriched in immune system processes, cell activation pathways, and cellular responses to lipids and fatty acid transport. For molecular function, the DEGs were predominantly enriched in enzyme binding, signalling receptor activity, and lipid binding. KEGG pathway analysis suggested that the anti-MAFLD effects of CFE were significantly linked to cytokine–cytokine receptor interaction, lipid and atherosclerosis, the AMPK signalling pathway, and the PPAR signalling pathway (Figure 4G). GSEA revealed that CFE negatively regulated pathways including lipid and atherosclerosis, cytokine–cytokine receptor interaction, MAPK signalling, and NF-κB signalling (Figure 4I; Supplementary Figure S2). On the positive enrichment side, CFE upregulated genes involved in the negative regulation of the PPAR signalling pathway and the AMPK signalling pathway (Figure 4J,K). Additional GSEA outcomes are presented in Supplementary Figure S2.

**Figure 5:**
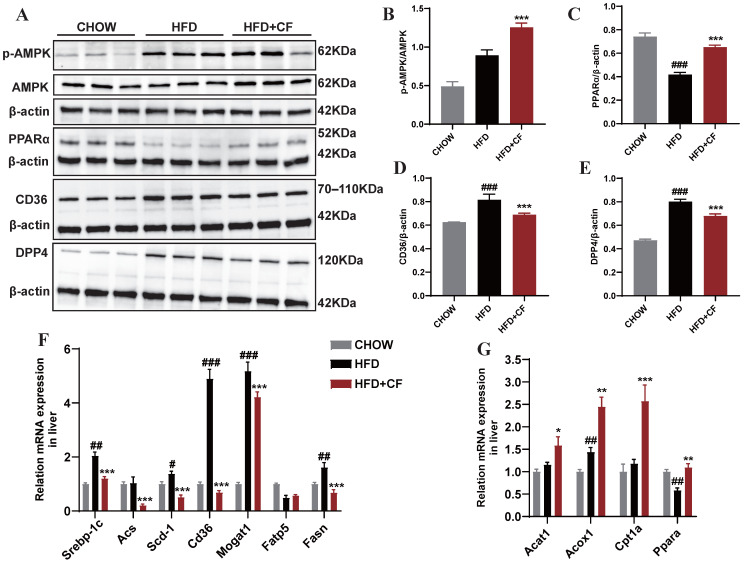
CFE regulates the AMPK/PPAR signalling pathway and lipid metabolism to alleviate MAFLD. (**A**) Western blot analysis of p-AMPK, AMPK, PPARα, CD36, and DPP4 protein expression levels in the liver. β-Actin was used as a loading control. (**B**–**E**) Quantitative analysis of relative protein abundance of p-AMPK/AMPK (**B**), PPARα (**C**), CD36 (**D**), and DPP4 (**E**) (*n* = 3 independent experiments). (**F**) Expression of lipid synthesis-related genes in the liver: *Srebp1c*, *Acs*, *Scd-1*, *Cd36*, *Mogat1*, *Fatp5* and *Fasn* mRNA (*n* = 6). (**G**) Expression of fatty acid oxidation-related genes in the liver: *Acat1*, *Acox1*, *Cpt1a* and *Pparα* mRNA (*n* = 6). All data are presented as means ± SD. Significant differences were determined by one-way ANOVA. # *p* < 0.05, ## *p* < 0.01, ### *p* < 0.001 vs. Chow group; * *p* < 0.05, ** *p* < 0.01, *** *p* < 0.001 vs. HFD group.

Subsequently, MAFLD-related genes were manually selected from the top 100 DEGs based on fold change, and a PPI network was constructed (Figure 4L). The results revealed high combined scores for PPARα, CD36, DPP4, Jun, CCL2, and FASN, indicating that CFE exerts its therapeutic effects through multiple targets. Among these core targets, PPARα and CD36 serve as key regulators in lipid metabolism, while DPP4 acts as a critical node linking glucose metabolism and inflammation. Western blot analysis showed that protein expression levels of p-AMPK and PPARα were reduced in the HFD group but significantly elevated following CFE treatment (Figure 5A–C; Supplementary Figure S3). In contrast, MAFLD mice exhibited markedly increased protein levels of CD36 and DPP4 (*p* < 0.001), which were reversed by CFE intake (*p* < 0.001; Figure 5D,E; Figure S3). Moreover, CFE intervention significantly downregulated hepatic lipid synthesis genes, including *Srebp1c*, *Acs*, *Scd-1*, *Cd36*, *Mogat1*, and *Fasn* (Figure 5F), while upregulating fatty acid oxidation-related genes such as *Ppara*, *Cpt1a*, *Acox1*, and *Acat1* (Figure 5G). Based on these results, we propose that modulation of the AMPK/PPAR signalling pathway may represent a potential mechanistic basis for the anti-MAFLD effects of CFE, although direct functional validation is still needed.

### 2.5. Correlation Analysis Among DEGs, DAMs, and Serological Indices in MAFLD

**Figure 6:**
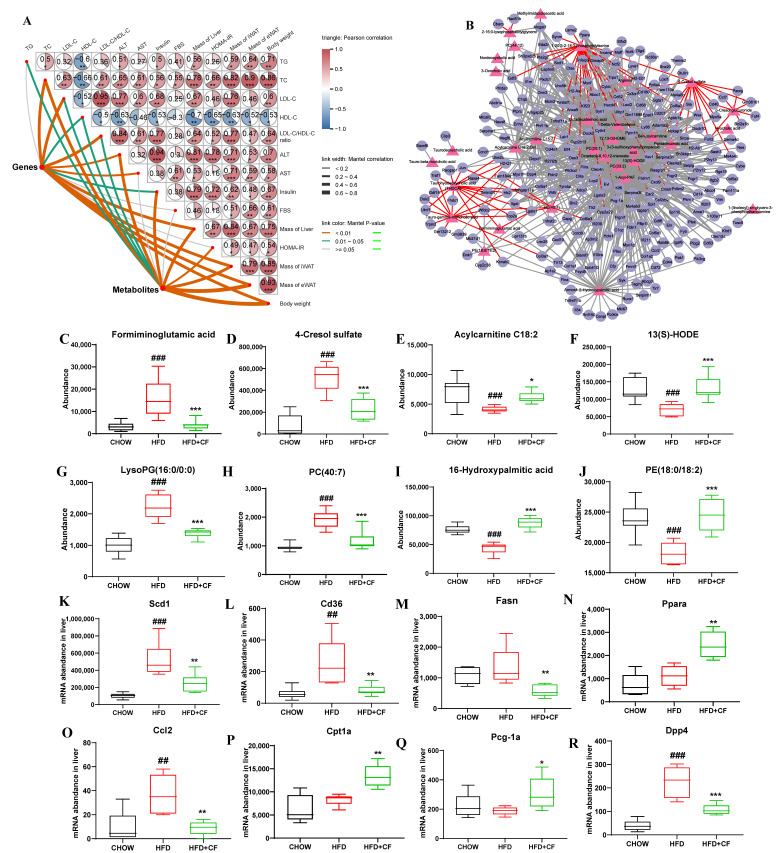
Integrative correlation analysis of DEGs, DAMs, and serological parameters in MAFLD. (**A**) Heatmap illustrating the correlation network among serological indicators, DEGs, and DAMs. (**B**) Correlation network between DEGs and DAMs (red edges indicate positive correlation; grey edges indicate negative correlation). (**C**–**J**) Relative abundance of representative metabolites in liver tissue. (**K**–**R**) Relative abundance of representative genes in liver tissue. For all correlation analyses, 95% confidence intervals (CIs) for correlation coefficients were calculated. All values are expressed as mean ± SD (*n* = 6). Statistical significance was assessed using one-way ANOVA. ## *p* < 0.01, ### *p* < 0.001 vs. chow group; *** *p* < 0.05, ** *p* < 0.01, * *p* < 0.001 vs. HFD group.

To better understand the contribution of CFE-associated metabolite and gene expression changes to MAFLD, a Spearman correlation matrix was constructed to systematically evaluate the association network among DAMs, DEGs, serum biochemical indicators, and phenotypic parameters. As shown in Figure 6A, serum lipid indices such as TC exhibited significant positive correlations with eWAT index (r = 0.90), iWAT index (r = 0.82), liver index (r = 0.78), and body weight (r = 0.86), suggesting a coordinated increase in adiposity and liver size. HDL-C showed negative correlations with atherogenic lipids (e.g., r = −0.52 with LDL-C, r = −0.66 with TC), consistent with its cardioprotective role. The LDL-C/HDL-C ratio, an important atherogenic index, was positively correlated with LDL-C (r = 0.95) and TC (r = 0.61). The liver index displayed moderate positive correlations with glycemic parameters such as insulin (r = 0.79) and HOMA-IR (r = 0.67), potentially linking hepatic steatosis to insulin resistance. Additionally, serum lipid profiles (e.g., TC and LDL-C) showed significant positive correlations with specific metabolites and genes. For TC, Mantel’s r was 0.51 for metabolites and 0.59 for genes; for LDL-C, Mantel’s r was 0.47 for metabolites and 0.66 for genes (all *p* < 0.01), suggesting that gene regulation in specific metabolic pathways may be associated with circulating lipid levels. Meanwhile, glucose metabolism indicators, represented by insulin and HOMA-IR, exhibited moderate to strong positive correlations with specific metabolites and genes. For insulin, Mantel’s r was 0.71 for metabolites and 0.49 for genes; for HOMA-IR, Mantel’s r was 0.27 for metabolites and 0.46 for genes (all *p* < 0.01), implying a potential link between hepatic metabolite accumulation and systemic insulin resistance.

Subsequently, Pearson correlation analysis was performed to assess associations between genes and metabolites. In the resulting correlation network, genes and metabolites are represented as circles and triangles, respectively, with red and grey edges indicating positive and negative correlations (Figure 6B). After retaining only strong (|r| > 0.8) and statistically significant (*p* < 0.05) correlations, the final metabolite–transcript network comprised 101 genes and 32 metabolites. Notably, most of these genes were enriched in the AMPK signalling pathway, PPAR signalling pathway and cytokine–cytokine receptor interaction. Key metabolites associated with these pathways included acylcarnitine C15:2, acylcarnitine C18:2, PC (20:1), and 12,13-DiHOME. To further validate these findings, we quantified the expression levels of the identified metabolites and genes in HFD-fed mice treated with CFE, with representative results shown in Figure 6C–R. CFE treatment significantly downregulated several metabolites, including formiminoglutamic acid, 4-cresol sulfate, acylcarnitine C18:2, lysoPG (16:0/0:0), and PC (20:1), while upregulating 13(S)-HODE, 16-hydroxypalmitic acid, and PE (18:0/18:2) (Figure 6C–J). Correspondingly, the expression of genes such as *Scd1*, *Cd36*, *Fasn*, *Pparα*, *Ccl2*, *Cpt1a*, *Pgc-1α*, and *Dpp4* was also significantly suppressed by CFE treatment (Figure 6K–R).

### 2.6. Screening Active Ingredients of CFE Anti-MAFLD

**Figure 7:**
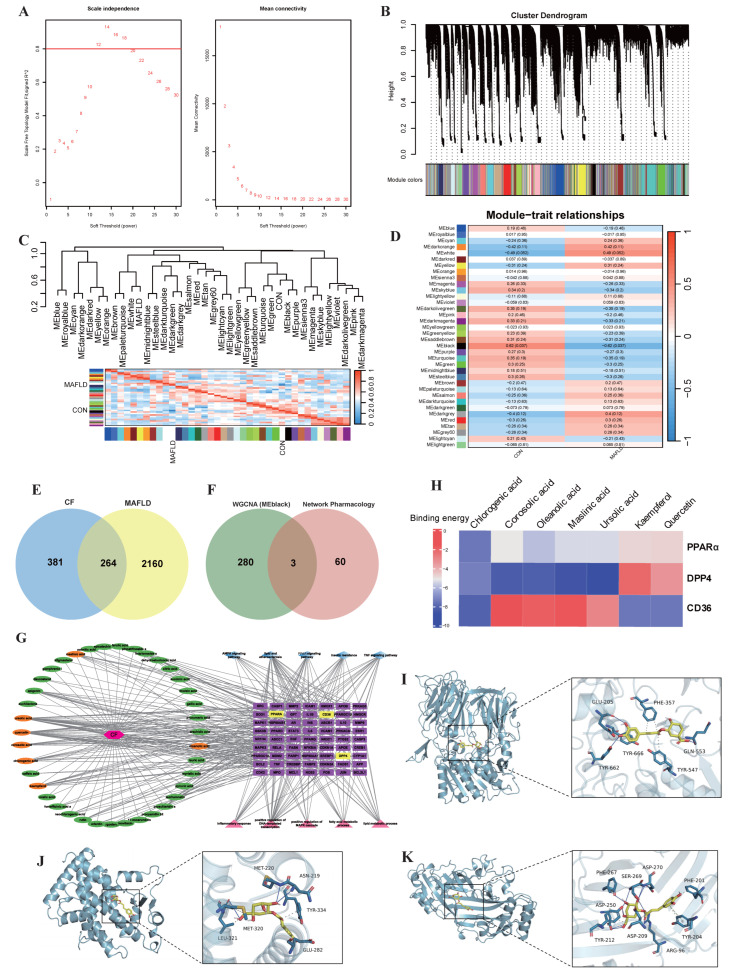
WGCNA and network pharmacology screening of potential active ingredients of CF against MAFLD. (**A**) Left panel: correlation coefficients at different power levels; right panel: average connectivity of the network constructed with each power value. (**B**) Gene cluster tree of MAFLD-related genes from the GSE183229 dataset. (**C**) Heatmap of expression values for all modules and bar charts of module eigengene values for each sample. (**D**) Correlation matrix between modules and MAFLD-related traits. (**E**) Venn diagram showing overlapping targets of CF and MAFLD identified by network pharmacology. (**F**) Venn diagram showing overlapping targets between WGCNA (MEblack module) and network pharmacology, highlighting three key targets: PPARα, DPP4, and CD36. (**G**) CF–ingredient–target–pathway network. (**H**) Heatmap of binding energies between PPARα, CD36, DPP4 and the seven active compounds from CF. (**I**–**K**) Binding conformations of CA in the active sites of PPARα (**I**), CD36 (**J**), and DPP4 (**K**), respectively.

WGCNA was used to identify gene modules highly relevant to specific phenotypes. According to the WGCNA report, selecting an appropriate power parameter for the adjacency matrix is crucial to satisfy the prerequisites of a scale-free network distribution. After testing power values from 1 to 30, a power of 12 was selected based on the scale free topology fit index (R^2^) and mean connectivity. As shown in Figure 7A, a power of 12 was the smallest value that achieved an R^2^ > 0.8 while maintaining a reasonable mean connectivity, indicating that the network approximated a scale free topology. Using this power value, a weighted co-expression network model was developed, classifying 5422 genes into 117 distinct modules (Figure 7B). Related modules were merged, resulting in 36 merged modules (Figure 7C,D). The correlation between module genes and clinical traits revealed that the ME-black module had correlation values exceeding 0.6. A total of 548 genes from this module were identified as most correlated with MAFLD.

To screened potential active components of CF, the TCMSP database was used to predict bioactive compounds and their targets. Based on established criteria, 54 active ingredients and 645 potential targets were selected. Overlapping these with MAFLD-associated disease targets yielded 264 shared drug–disease targets (Figure 7E). KEGG and GO enrichment analyses of these intersection targets are provided in Supplementary Figure S4. A comprehensive CF–ingredient–target–pathway network was constructed, integrating 38 CF constituents with 63 core proteins involved in pathways such as AMPK signalling, lipid metabolism and atherosclerosis, and PPAR signalling (Figure 7G). Venn diagram analysis comparing genes from the ME-black module with network pharmacology-derived and CF-predicted targets identified three overlapping candidate key targets: PPARα, DPP4, and CD36 (Figure 7F). Their associated potential active components included chlorogenic acid (CA), ursolic acid (UA), oleanolic acid (OA), maslinic acid (MA), corosolic acid (CSA), quercetin, and kaempferol.

The potential binding between active compounds and core targets was predicted computationally using AutoDock Vina. Molecular docking was subsequently carried out for three candidate targets (PPARα, DPP4, and CD36) and seven potential active ingredients. The binding energies of these target proteins are presented in Supplementary Table S3, and a corresponding heatmap is shown in Figure 7H. In principle, a lower ligand–receptor binding energy indicates a more stable conformation, with −5.0 kcal/mol typically considered the threshold. Taking CA as an example (Figure 7I–K), CA interacts with PPARα through hydrogen bonds involving ASN219, MET220, and MET320, as well as hydrophobic interactions with GLU282, MET320, LEU321, and TYR334 (Figure 7J). For DPP4, CA forms hydrogen bonds with GLU205, GLN553, and TYR662, alongside hydrophobic contacts with PHE357, TYR547, and TYR666 (Figure 7I). Regarding CD36, CA engages via hydrogen bonds with ARG96, TYR212, ASP250, PHE267, SER269, and ASP270, complemented by hydrophobic interactions with PHE201, TYR204, ASP209, and ASP250 (Figure 7K). Collectively, these results suggest that CA, UA, OA, MA, and CSA exhibit favorable predicted binding energies with their respective target proteins, providing theoretical support for further experimental validation. LC-MS confirmed the presence of these five components in the CF extract (Supplementary Figure S6, Table S2).

### 2.7. Active Ingredients Improve Lipid Levels in THLE-2 Cells

**Figure 8:**
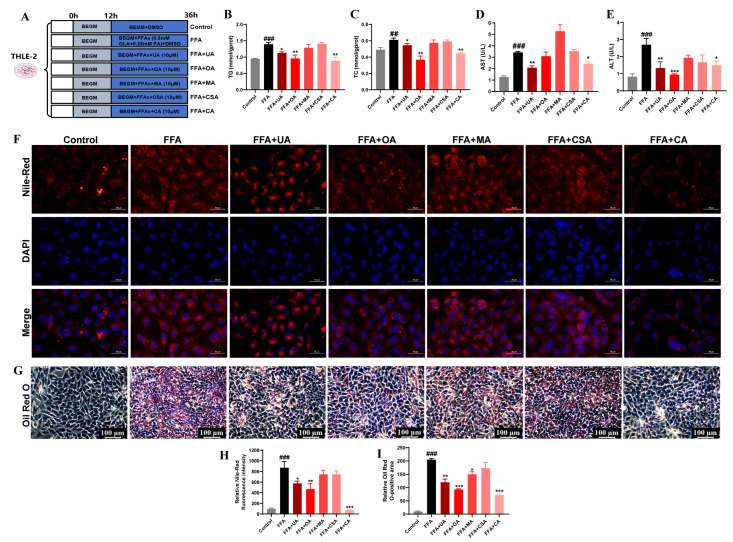
Effects of UA, OA, MA, CSA, and CA on FFA-induced lipid accumulation in THLE-2 cells. (**A**) Schematic workflow of the cell culture experiment. THLE-2 cells were exposed to FFAs and simultaneously treated with 10 μM of UA, OA, MA, CSA, or CA for 24 h. The experiment was performed in triplicate. (**B**,**C**) Intracellular concentrations of TG and TC. (**D**,**E**) Concentrations of AST and ALT in the cell culture supernatant. (**F**) Representative Nile Red staining images (red, microtubules) and (**H**) corresponding relative red fluorescence intensity. Magnification: ×400; scale bar: 50 μm. (**G**) Representative Oil Red O staining images and (**I**) relative Oil Red O-positive area. Images were captured under a light microscope using a ×20 objective and ×10 eyepiece. Scale bar: 100 μm. All data are expressed as mean ± SD (*n* = 3). Statistical significance was evaluated using one-way ANOVA. ## *p* < 0.01, ### *p* < 0.001 vs. control group; * *p* < 0.05, ** *p* < 0.01, *** *p* < 0.001 vs. FFA group.

We next evaluated the effects of CA, UA, OA, MA, and CSA on FFA-induced lipid deposition in hepatocytes (Figure 8A). The optimal FFA concentration for inducing lipid deposition in THLE-2 cells was first determined (Supplementary Figure S7F). Based on a comprehensive assessment of lipid accumulation and cell viability, 0.75 mM FFA was selected for subsequent experiments. As shown in Supplementary Figure S7A–E, a CCK-8 assay assessed the effects of different concentrations (10, 25, 50, 100, and 200 μM) of each monomer on THLE-2 cells after 24 h. Based on cell viability, the optimal working concentrations were determined to be 10 μM for OA, UA, MA, CSA, and CA. Dose–response analyses of intracellular TC and TG levels demonstrated that CA, UA, and OA exhibited a favourable dose-dependent effect at 5 μM and 10 μM, with a more pronounced lipid-lowering effect observed at 10 μM (Figure S7G,H). As shown in Figure 8B,C, FFA stimulation significantly increased intracellular TC and TG concentrations (*p* < 0.01 vs. control), while treatment with CA, UA, and OA markedly reversed these elevations (*p* < 0.01 vs. FFA-induced cells). Furthermore, FFA-induced hepatocyte injury was reflected by significantly elevated AST and ALT levels in the cell culture supernatant. Treatment with UA or CA significantly attenuated this damage, markedly reducing the release of both enzymes (Figure 8D,E). In contrast, although OA effectively lowered ALT levels, it did not significantly affect AST release. Consistent with these findings, Nile Red (Figure 8F,H) and Oil Red O (Figure 8G,I) staining further demonstrated that CA, UA, and OA treatment significantly alleviated FFA-induced lipid deposition in THLE-2 cells (*p* < 0.01 vs. FFA-induced cells). Collectively, these data indicate that CA, UA, and OA are the primary active constituents in CFE responsible for ameliorating MAFLD.

## 3. Discussion

Current evidence suggests that poor dietary habits and lifestyle factors are the main drivers of obesity and metabolic dysfunction, both of which are major triggers of MAFLD. MAFLD is recognised as the most common chronic liver disease, affecting about 30% of the adult population, with approximately 20% of cases classified as metabolic dysfunction-associated steatohepatitis (MASH) [22]. Preventing MAFLD progression underscores the need to effectively manage metabolic abnormalities. However, existing treatment strategies are complex and show limited efficacy. Over the past two decades, traditional Chinese medicine (TCM) has drawn increasing attention for its ability to regulate metabolism and alleviate fatty liver disease through multiple mechanisms, including inhibition of triglyceride synthesis, modulation of fatty acid absorption and transport, and enhancement of lipophagy. CF, a widely used medicinal and edible product, features a “dietary therapy” nature that greatly improves long-term treatment adherence, which is well suited to the lifelong management required for MAFLD. Although CF is commonly used to prevent and treat obesity and hyperlipidaemia, its active ingredients and mechanism of action in MAFLD therapy still warrant further investigation.

In this study, a long-term HFD was used to induce pathology in C57BL/6J mice, which closely recapitulates the pathogenesis of MAFLD [23,24]. The HFD-fed mice developed severe lipid deposition accompanied by significant insulin resistance. Lipid accumulation led to visible obesity, a major driver of MAFLD [25]. Furthermore, excessive lipid deposition is known to trigger inflammatory responses [26]. It is well established that saturated fatty acids directly stimulate the expression of inflammatory genes via Toll-like receptor 4 (TLR4) signalling [27]. In addition, the lipid-laden liver develops insulin resistance, which is commonly associated with chronic low-grade inflammation, further promoting MAFLD progression [28]. Our study reveals that CFE exerts a profound regulatory effect on lipid metabolism in MAFLD model mice, independent of changes in caloric intake. This finding suggests a possible increase in energy expenditure or thermogenesis, which would contribute to weight loss. However, because we did not perform metabolic cage measurements or assess brown adipose tissue activity in this study, this hypothesis cannot be directly confirmed. The marked reduction in white adipose tissue mass indicates a substantial alteration in lipid homeostasis, suggesting enhanced lipid catabolism or suppressed lipogenesis in adipose tissue. Concurrently, the significant decreases in liver mass and lipid content, together with reduced serum concentrations of TG, TC, and LDL-C, point to enhanced hepatic lipid clearance and reduced ectopic lipid accumulation, reflecting more efficient systemic lipid metabolism. The parallel reductions in ALT and AST activities provide further evidence of diminished hepatocellular injury, likely secondary to the alleviation of hepatic steatosis. However, we did not assess liver fibrosis (e.g., by collagen staining) or quantify inflammatory cells, which are important for evaluating progression to steatohepatitis. Future studies should incorporate these assessments to better characterise the effects of CFE on liver pathology. Improvements in insulin resistance were evidenced by decreased glucose tolerance test (GTT), insulin tolerance test (ITT), and homeostatic model assessment for insulin resistance (HOMA-IR) values. The amelioration of insulin resistance is closely associated with the restoration of a healthy lipid balance, achieved through the regulation of lipid levels in both blood and liver. Collectively, these findings indicate that CFE possesses the capacity to modulate systemic lipid metabolic flux and holds potential for application in the management of MAFLD, a condition characterised by disordered lipid metabolism and hepatic lipid accumulation.

Metabolomics, a promising approach for investigating small-molecule metabolites, has been widely applied in MAFLD research. In this study, we conducted serum untargeted metabolomic analysis across the normal, HFD, and CF_H groups. The results showed that the majority of differentially expressed metabolites were lipids, fatty acids, bile acids, and amino acids, and that CFE reversed these aberrantly expressed metabolites toward normal physiological levels. Bile acids regulate hepatic lipid and glucose homeostasis as well as energy metabolism, processes closely associated with MAFLD [29]. Studies have shown that individuals with MAFLD exhibit significantly elevated levels of primary bile acids (such as taurocholic acid, TCA) and secondary bile acids (such as taurodeoxycholic acid, TDCA) [30], which is consistent with the findings of this study (Supplementary Data S1). Acylcarnitines are fatty acid metabolites that serve as shuttles during fatty acid β-oxidation, facilitating the transport of fatty acids into mitochondria for energy production [31,32]; their levels influence MAFLD progression [33]. Acylcarnitine C18:2 was markedly elevated in the MAFLD model, whereas its levels were significantly reduced following CFE treatment. Lysophosphatidylcholine (LPC) is a bioactive proinflammatory lipid generated under pathological conditions and plays a critical role in fatty acid delivery [34]. Elevated total serum phosphatidylcholine (PC) content is a prominent feature in patients with MAFLD [35], the increases in PC (20:1) and LPC (20:1) observed in the model group were both reversed by CFE treatment, suggesting that CFE may alleviate hepatic lipid metabolic disorders by modulating PC metabolism and promoting cellular lipid homeostasis. In addition, perturbations in amino acid metabolism, such as that of tyrosine, also influence MAFLD. Pathway enrichment analysis of differentially accumulated metabolites (DAMs) revealed that CFE may exert its ameliorative effects on MAFLD by modulating metabolic pathways including fatty acid biosynthesis, secondary bile acid biosynthesis, and the PPAR signalling pathway.

To further investigate the mechanism underlying the effects of CFE on lipid metabolism, we performed transcriptomic analyses. RNA sequencing of liver tissues revealed that HFD significantly upregulated the gene expression of CD36 [36], SCD1 [37], FASN [38], and DPP4 [39], while downregulating PGC-1α and PPARα. HFD downregulated most genes (4208 down vs. 381 up), consistent with HFD-induced metabolic dysfunction, reflecting inhibition of fatty acid oxidation, oxidative phosphorylation, and insulin signalling pathways. CFE administration markedly reversed these changes. Integrated enrichment analysis using KEGG and GSEA identified pathways related to lipid and atherosclerosis, the AMPK signalling pathway, and the PPAR signalling pathway as the primary pathways potentially involved in the regulation of lipid metabolism by CF to alleviate MAFLD. AMPK is a major energy sensor that plays critical regulatory roles in metabolic diseases, inflammation, fibrosis, and cancer, and its activity is abnormally reduced in MAFLD [40]. Notably, despite the absence of p-ACC measurements, qPCR analysis indirectly corroborated AMPK activation by revealing that CFE significantly suppressed the expression of *Srebp1c*, *Fasn*, and *Scd1*, which are key genes governing hepatic fatty acid and triglyceride synthesis. PPARs can respond to the regulation of AMPK and belong to a class of ligand-activated transcription factors, including PPARα, PPARβ/δ, and PPARγ isoforms. PPARα is primarily expressed in the liver and is involved in fatty acid oxidation and metabolic pathways, thereby contributing to lipid and energy homeostasis [41]. PGC-1α serves as a transcriptional co-activator of PPARα, and their interaction activates downstream mitochondrial fatty acid oxidation. The role of CD36 is context-dependent; it can induce co-activation of PPARα in hepatocytes via PGC-1α signalling and indirectly influence fatty acid oxidation [42]. However, under chronic HFD conditions, CD36 upregulation is often associated with increased lipid influx and exacerbation of steatosis. DPP4 is a serine protease that degrades glucagon-like peptide-1 (GLP-1), a derivative of proglucagon that plays a critical role in glycemic regulation and energy metabolism. Multiple studies have demonstrated that hepatic DPP4 mRNA and protein expression is significantly elevated in patients with MAFLD compared with normal controls. Research indicates that DPP4 is a target gene of CCL2 and NF-κB [43], and its expression is associated with elevated levels of the inflammatory cytokines IL-1β, TNF-α, and IL-6 [44]. Therefore, reducing DPP4 protein expression may alleviate MAFLD by improving insulin resistance and attenuating inflammation. Correlation analysis of DAMs, DEGs, and MAFLD phenotypic indicators revealed complex interrelationships among these variables. A network integrating DAMs and DEGs was constructed to explore potential regulatory relationships between metabolic phenotypic changes and gene expression alterations. The results showed that the expression of key target genes, including *Pparα*, *Cd36*, *Dpp4*, and *Ccl2*, was associated with the metabolism of 13(S)-HODE, acylcarnitine C18:2, and PC (20:1).

By harnessing data from the Gene Expression Omnibus (GEO) database, we identified abnormally expressed genes associated with MAFLD. Network pharmacology predicted potential active components and therapeutic targets of CF. Integration of WGCNA-derived MAFLD-related genes with network pharmacology targets revealed three critical target genes, namely PPARα, DPP4 and CD36, through which CF exerts its therapeutic effects. Five components, including CA, UA, OA, MA and CSA, were identified in association with these targets (Figure 7). Given that hepatic lipid accumulation is a key driver of MAFLD, we used FFA-induced THLE-2 cells to model lipid deposition and evaluated the lipid-lowering effects of these five components. Among them, CA, UA and OA exhibited superior efficacy compared with MA and CSA. Previous studies have shown that CA alleviates autophagy and insulin resistance by suppressing the JNK pathway in a rat model of MAFLD [45]; UA suppresses MAFLD by regulating lipid metabolism and attenuating endoplasmic reticulum stress; and OA ameliorates MAFLD by rebalancing the gut–liver axis homeostasis through modulation of endotoxin-mediated TLR4-related pathways. These findings support the reliability of our cellular results and suggest that CA, UA and OA are the active constituents of CF against MAFLD. It should be noted, however, that these cell-based experiments were designed to provide complementary phenotypic support for the multi-omics mechanistic findings; they did not assess key signalling molecules such as AMPK, PPARα, CD36 or DPP4 activity. Nevertheless, the results lay a foundation for subsequent in vivo studies to further validate the underlying mechanisms.

## 4. Materials and Methods

### 4.1. Materials and Reagents

LC/MS-grade acetonitrile (ACN), methanol, isopropyl alcohol (IPA), and acetic acid were purchased from Fisher Chemical China Branch (Shanghai, China). LC/MS-grade ammonium acetate was obtained from Sigma-Aldrich (St. Louis, MO, USA). Biochemical assay kits for TG, TC, LDL-C, HDL-C, AST, ALT, as well as the cell counting kit-8 (CCK8), were all supplied by Nanjing Jiancheng Institute of Bioengineering (Nanjing, China). D-Glucose (≥99.5%) and human insulin were products of Sigma-Aldrich (St. Louis, MO, USA). A blood glucose meter and corresponding test strips were obtained from Roche Diagnostic GmbH (Accu-Chek Active, Mannheim, Germany). The ultrasensitive mouse insulin ELISA kit was provided by Shanghai Enzyme-Linked Biotechnology Co., Ltd. (Shanghai, China). The HFD was sourced from Research Diets Co., Ltd. (D09100310, New Brunswick, NJ, USA). Reference standards for CA, UA, OA, MA, and CSA were purchased from Shanghai Stant Standard Technical Services Co., Ltd. (Shanghai, China).

### 4.2. Preparation of CF Extract and HPLC/Q-TOF-MS Analysis

*Crataegi fructus* (batch No. 20241204) was obtained from Anjia Pharmaceutical Co., Ltd. (Anguo, China) and subjected to chemical analysis in accordance with the Chinese Pharmacopoeia, which confirmed its satisfactory quality. The crude drug was morphologically identified by Professor Yuan Yuan from the Institute of Chinese Materia Medica, China Academy of Chinese Medical Sciences. Voucher specimens (Voucher No. CF-001 to CF-006) have been deposited in the authors’ laboratory. Briefly, the fruits were soaked in 95% ethanol at a liquid-to-solid ratio of 10:1 (*v*/*w*) for 2 h. The mixture was then decocted for a further 2 h and filtered through gauze. The residue was re-extracted with 95% ethanol at a liquid-to-solid ratio of 6:1 under the same conditions. The combined filtrates were centrifuged at 3000 rpm for 15 min, and the supernatant was evaporated under vacuum using a rotary evaporator (OSB-2200, EYELA, Tokyo, Japan). The concentrated extract was carefully transferred into a vacuum freeze-dryer (4LSC plus, Christ Alpha, Benningen, Germany). After freeze-drying for 3 days (−60 °C, 0.1 mbar), the lyophilized powder of CF extract was obtained, with an extraction yield of approximately 18.5% (*w*/*w*).

For LC-MS analysis, the freeze-dried powder was redissolved in methanol, ultrasonically treated to ensure complete dissolution, and then passed through a 0.22 μm filter membrane. The final concentration for analysis was adjusted to 0.1 g/mL. CFE components were identified using an XionLC X500R QTOF system (AB Sciex, Marlborough, MA, USA) fitted with an ACQUITY UPLC HSS T3 column (100 × 2.1 mm, 1.8 μm; Waters, Milford, MA, USA). The same LC-MS conditions were applied for both positive and negative ionization modes, as detailed in Supplementary Material Figure S5 and Table S1.

### 4.3. Animals and Treatment

Male C57BL/6J mice (6 weeks old) were obtained from Wuhan Hualianke Biotechnology Co., Ltd. (Wuhan, China, Certificate No. SCXK (E) 2023-0033) and housed in the SPF facility of the Experimental Animal Center at the Jiangxi Health Industry Institute of Traditional Chinese Medicine. All animal experiments were approved by the CACMS Ethics Committee (Approval No. 2025011), and every effort was made to minimize pain and distress. A total of 60 mice were kept under standard conditions: temperature 22 ± 2 °C, humidity 60–70%, and a 12 h light/dark cycle. They were randomly housed in groups of three to five with free access to water and food. After one week of acclimation, 10 mice were placed on a normal chow diet (CHOW group), while the remaining 50 were fed a HFD (D09100310) for 10 weeks to induce hepatic steatosis. The HFD consisted of 40% fat, 20% fructose, and 2% cholesterol. Following HFD feeding, the obese mice were randomly assigned to five groups (n = 10 each): HFD alone, HFD supplemented with daily oral doses of CF at 0.3 g/kg (HFD+CF_L), 0.6 g/kg (HFD+CF_M), or 1.2 g/kg (HFD+CF_H), and HFD plus 30 mg/kg obeticholic acid (HFD+OCA). The CF doses were selected based on prior laboratory experience and confirmed to be safe [46]. The 10-week HFD feeding successfully established a reliable MAFLD mouse model. Body weight and food intake were recorded weekly.

### 4.4. Glucose Tolerance and Insulin Tolerance Tests

For the glucose tolerance test (GTT), mice underwent a 14 h fasting period, and baseline fasting glucose levels were measured from tail vein blood. Afterwards, glucose (2 g/kg) was administered via intraperitoneal injection, and blood glucose levels were recorded at 15, 30, 45, 60, and 120 min post-injection. For the insulin tolerance test (ITT), mice were fasted for 5 h before receiving an intraperitoneal injection of insulin (0.75 U/kg). Tail blood glucose concentrations were then measured at the same time intervals (15, 30, 45, 60, and 120 min after injection).

### 4.5. Collection and Preparation of Samples

At the conclusion of the experiment, all animals were fasted for 12 h. Mice were anesthetized with isoflurane, and blood was collected via cardiac puncture. Immediately afterward, inguinal white adipose tissue (iWAT), epididymal white adipose tissue (eWAT), brown adipose tissue (BAT), and the liver were freshly excised and weighed. A portion of each tissue was fixed in 4% paraformaldehyde for later analysis, while the remaining tissue was stored at −80 °C. The whole blood samples were left to clot at room temperature for 4 h, then centrifuged at 3500 rpm for 15 min. The resulting serum was aliquoted and kept at −80 °C for future use.

### 4.6. Biochemical Parameters Analysis

To evaluate glucose metabolism, mice were fasted for 12 h, followed by collection of 3 μL of blood from the tail vein. Fasting blood glucose (FBG) levels were determined using a glucose meter. Serum fasting insulin (FINS) concentrations were measured with an ELISA kit. The insulin resistance index (HOMA-IR) was calculated using the formula: HOMA-IR = FBG (mM) × FINS (IU/L)/22.5. Serum levels of TC, TG, LDL-C, HDL-C, AST, and ALT were assessed with a multifunctional microplate reader (Victor Nivo, Waltham, MA, USA) according to the manufacturer’s instructions. Hepatic contents of TG and TC were also quantified.

### 4.7. Histological Examination and Assessment

H&E and Oil Red O staining of liver and adipose tissues were carried out by Servicebio Biotechnology Co., Ltd. (Wuhan, China) according to established protocols [47]. For each tissue, five clear and intact sections were examined and imaged under a light microscope (PANNORAMIC MIDI II, 3D HISTECH Kft., Budapest, Hungary). Histological evaluation of liver samples was performed using the MAFLD Activity Score (MAS), a semi-quantitative scoring system developed by the U.S. Nonalcoholic Steatohepatitis Clinical Research Network (NASH-CRN) [48]. For histological analysis, sample sections were coded by an independent researcher, and two pathologists blinded to the group allocation performed NAS scoring and quantified Oil Red O staining. Image analysis was conducted using ImageJ software by an investigator blinded to the treatment groups.

### 4.8. Non-Targeted Metabolomics Analysis

#### 4.8.1. Extraction of Metabolites

Serum samples (100 μL) were placed into EP tubes and reconstituted with 400 μL of ice-cold methanol–acetonitrile (1:1, *v*/*v*) spiked with internal standards (200 μg/mL: d3-leucine and d6-phenylalanine). The mixtures were vortexed for 60 s and then sonicated for 15 min. Protein precipitation was achieved by incubating the samples at −20 °C for 1 h, followed by centrifugation at 13,000 rpm for 15 min at 4 °C. The resulting supernatants were collected and evaporated to dryness under vacuum centrifugation. The dried residues were redissolved in 100 μL of 80% ACN to prepare the test solutions, which were subsequently injected into the LC-MS/MS system. Quality control (QC) samples were generated by mixing 10 μL from each individual sample. Blank samples were processed in the same manner but without the addition of internal standards.

#### 4.8.2. LC-MS Analyses

Data acquisition was carried out using an ultra-high-performance liquid chromatography (UHPLC) system coupled with a XionLC-X500R QTOF mass spectrometer (AB Sciex, USA). LC separation was performed on an ACQUITY UPLC BEH Amide column (1.7 µm, 100 mm, Waters, USA) and a Kinetex^®^ C18 column (2.6 µm, 2.1 × 100 mm, Phenomenex, Torrance, CA, USA), both maintained at 25 °C. For hydrophilic interaction liquid chromatography (HILIC), mobile phase A consisted of 25 mM NH_4_OH and 25 mM NH_4_OAc in water, while mobile phase B was ACN for both positive and negative ionization modes. The flow rate was 0.5 mL/min, with the gradient program: 0–0.5 min, 95% B; 0.5–7 min, 95–65% B; 7–8 min, 65–40% B; 8–9 min, 40% B; 9–9.1 min, 40–95% B; 9.1–12 min, 95% B. The injection volume was 2 µL. For reverse-phase liquid chromatography (RPLC), mobile phase A was 0.01% acetic acid in water, and mobile phase B was IPA: ACN (1:1, *v*/*v*) for both polarities. The flow rate was 0.3 mL/min, using the following gradient: 0–1 min, 1% B; 1–8 min, 99% B; 8–9 min, 99% B; 9.0–9.1 min, 99–1% B; 9.1–12 min, 1% B. All samples were injected in a random order.

Mass spectrometry was performed in full-scan mode with polarity switching between positive and negative ions. MS/MS spectra were acquired using information-dependent acquisition (IDA). The source conditions were gas 1, gas 2, curtain gas, collision-activated dissociation (CAD) gas, and temperatures set to 50 psi, 50 psi, 35 psi, 7 psi, and 500 °C, respectively. Spray voltages were 5500 V (positive) and −4500 V (negative). Mass scan ranges were 100–1500 Da for MS and 50–1500 Da for MS/MS. The declustering potential (DP) was fixed at 60 V, while collision energy (CE) was 10 V for MS and 25 ± 15 V for MS/MS. The top ten ions with intensities ≥ 100 were automatically selected as precursors to trigger IDA-based MS/MS, with dynamic background subtraction enabled. Dynamic exclusion was set to 3.0 s, and isotope exclusion was activated.

#### 4.8.3. Metabolomics Annotation Processing

Raw MS data (.wiff) were transformed into .mgf format using ProteoWizard (v3.2, ProteoWizard Software Foundation, Palo Alto, CA, USA). Peak detection, retention time correction, and alignment were performed with MS-DIAL v5.3.240704 (Tokyo University of Agriculture and Technology, Japan) [49]. Metabolite annotation was carried out using MetDNA v1.2.2 (http://metdna.zhulab.cn/) [50]. Parameters were set as ‘HILIC’ or ‘RP’ according to the LC mode, and collision energy was set to ‘30’. Only metabolites with confidence levels 1, 2, or 3.1 were retained for subsequent analyses. Data points with intensity values exceeding five standard deviations were considered outliers and treated as missing values, which were then imputed using the K-nearest neighbor (KNN) algorithm. All metabolites were normalized to a standard normal distribution (mean = 0, SD = 1) prior to statistical analysis.

#### 4.8.4. Data Statistical Analysis

PCA and OPLS-DA were conducted using the Metware Cloud Platform (https://cloud.metware.cn/#/tools/, accessed on 1 December 2025), an integrated tool for metabolomics data processing. Univariate analysis (*t*-test) was used to calculate both the statistical significance (*p*-value) and fold change (FC) for each metabolite between groups. Differential accumulated metabolites (DAMs) were defined as those with variable importance in projection (VIP) > 1.0, |log_2_FC| > 0.3, and −log_10_(*p*-value) > 1.3. Volcano plots generated by the same platform were employed to screen metabolites of interest based on |log_2_FC|, −log_10_(*p*-value), and VIP values. The identified DAMs were then subjected to cluster analysis, and heatmaps were constructed after Z-score normalization of differential metabolite intensities, also using the Metware Cloud Platform. Finally, GO and KEGG enrichment analyses of the DAMs were performed with Metascape (https://metascape.org/, accessed on 20 December 2025).

### 4.9. RNA-Sequencing Analysis

Total RNA was isolated from fresh liver samples using TRIzol reagent. RNA quantity and integrity were assessed with an RNA Nano 6000 Assay Kit on a Bioanalyzer 2100 system (Agilent Technologies, Santa Clara, CA, USA). This purified RNA was then used as the starting material for library preparation. mRNA enrichment involved several steps: capture with poly-T-coated magnetic beads, fragmentation, reverse transcription, end repair, A-tailing, adapter ligation, purification, and PCR amplification. Library fragments were further purified using the AMPure XP system (Beckman Coulter, Brea, CA, USA), and final library quality was verified on an Agilent 2100 Bioanalyzer. Qualified libraries were sequenced on an Illumina NovaSeq 6000 platform. PCA was employed to assess sample clustering, and DESeq2 was used to identify differentially expressed genes (DEGs) under the criteria of −log_10_(*p*-value) > 1.3 and |log_2_FC| > 0.5. Volcano plots and heatmaps were generated using the Metware Cloud Platform. GO and KEGG enrichment analyses of DEGs (*p* < 0.05) were performed with OmicShare Tools (http://www.omicshare.com/tools, accessed on 16 February 2026), and the resulting plots were visualized via the Metware Cloud Platform.

### 4.10. Gene Set Enrichment Analysis

Gene Set Enrichment Analysis (GSEA) was performed using the Omicshare Tools platform (http://www.omicshare.com/tools, accessed on 25 December 2025). Genes were ranked by the log_2_ fold change derived from DESeq2 differential expression analysis comparing the HFD+CF_H vs. HFD groups, as well as the CHOW vs. HFD groups. The Molecular Signatures Database (MSigDB) gene sets were used as reference collections, including the Hallmark gene sets (h.all.v2023.1.Hs.symbols.gmt) and the C5 Gene Ontology (GO) biological process gene sets. Enrichment was assessed using 1000 permutations with the weighted enrichment statistic. Significantly enriched gene sets were defined by a normalized enrichment score (NES) with |NES| > 1 and an FDR-adjusted *p*-value < 0.25, which is the default significance threshold for GSEA.

### 4.11. Identification of MAFLD-Related Genes Based on WGCNA

Publicly available datasets from the NCBI Gene Expression Omnibus (GEO) database (https://www.ncbi.nlm.nih.gov/geo/, accessed on 22 December 2025) were screened based on the following criteria: (1) transcriptomic datasets containing both MAFLD and normal human liver samples; (2) data derived from *Homo sapiens*; (3) complete sample information without missing values. The GSE183229 dataset (based on the GLP20301 platform) was chosen for subsequent analysis. Raw GEO data were normalized using the ‘Normalize Arrays’ function in R (v4.4.2, RStudio Software, Boston, MA, USA). WGCNA was carried out with the same R package to identify modules correlated with MAFLD and to extract MAFLD-related genes.

### 4.12. Network Pharmacology Analysis

The chemical constituents of CF were retrieved from the TCMSP (https://tcmsp-e.com/tcmsp.php, accessed on 20 December 2025) and ITCM (http://itcm.biotcm.net/index.html, accessed on 22 December 2025) databases. Corresponding protein targets of these components were obtained from TCMSP, SwissTargetPrediction (http://www.swisstargetprediction.ch/, accessed on 1 January 2026), and DrugBank (http://www.drugbank.ca/, accessed on 1 January 2026). MAFLD-related targets were sourced from GeneCards (https://www.genecards.org/, accessed on 1 January 2026), DisGeNET (https://disgenet.com/, 1 January 2026), and TTD (https://db.idrblab.net/ttd/, accessed on 1 January 2026). Only the overlapping targets between component-associated and disease-associated sets were kept for further analysis. A protein–protein interaction (PPI) network was built using the STRING database (https://string-db.org/, 19 January 2026), and targets with a degree value below 10 were excluded to prioritize highly connected nodes. Functional enrichment analysis was conducted on the DAVID platform (https://davidbioinformatics.nih.gov/, accessed on 19 January 2026) with a significance cutoff of *p* < 0.01. Finally, an integrated four-node network (herbs, components, targets, pathways) was constructed and visualized using Cytoscape v3.10.2 (Cytoscape Software, San Diego, CA, USA).

### 4.13. Molecular Docking

Compound structures were retrieved from the PubChem database (https://pubchem.ncbi.nlm.nih.gov/) and converted from SDF to PDB format using Open Babel v2.3.2. Receptor protein structures were obtained from the Protein Data Bank (PDB) (https://www.rcsb.org/). Before docking, receptor proteins were preprocessed in PyMOL v2.3.4 to remove water molecules and any bound ligands. Further preparation steps—such as adding hydrogen atoms and balancing charges—were carried out with AutoDockTools. Both receptors and ligands were then converted into PDBQT format. Molecular docking was performed using AutoDock Vina v1.1.2, and the resulting complexes were analysed using the Protein–Ligand Interaction Profiler (PLIP). Docking outcomes were visualized with PyMOL.

### 4.14. Cell Culture and Treatment

The human hepatic cell line THLE-2 (batch ZQ1033) was acquired from Shanghai Zhong Qiao Xin Zhou Biotechnology Co., Ltd. (Shanghai, China). Cells were maintained in THLE-2 complete medium (batch ZQ-1340) and routinely tested for mycoplasma contamination. For cytotoxicity assays, cells were seeded at 1.0 × 10^4^ per well in 96-well plates. UA, OA, MA, CSA, and CA were prepared in DMSO at concentrations of 200, 100, 50, 25, and 10 μM. After 24 h of treatment, cell viability was evaluated using the CCK-8 kit.

For pharmacodynamic assessment, cells were plated at 2 × 10^5^ per well in 6-well plates. Upon reaching 80% confluence, steatosis was induced by exposure to an FFA mixture (oleic acid 0.5 mM, palmitic acid 0.25 mM, ratio 2:1) for 24 h. Cells were then divided into the following groups: control, FFA model, FFA + UA (10 μM, 5 μM), FFA + OA (10 μM, 5 μM), FFA + MA (10 μM, 5 μM), FFA + CSA (10 μM, 5 μM), and FFA + CA (10 μM, 5 μM). Following another 24 h, 20 μL of supernatant from each group was collected for AST and ALT measurements. Cells were subsequently harvested to determine TG and TC levels, and lipid deposition was assessed using Nile Red and Oil Red O staining.

### 4.15. Western Blot Analysis

Liver tissue proteins were extracted following established protocols. Samples (80 μL each) were mixed with 20 μL of loading buffer and boiled for 10 min to denature proteins. After SDS-PAGE separation by molecular weight, proteins were semi-dry transferred onto PVDF membranes. Membranes were blocked with skim milk for 1 h at room temperature, then incubated overnight with primary antibodies. A fluorescent secondary antibody (#SAB48169, Bioswamp, Wuhan, China) was applied for 1 h at room temperature in the dark. Target protein levels (AMPK, p-AMPK, CD36, DPP4, and PPARα) were detected using a ChemiDoc imaging system (Bio-Rad Laboratories, Inc., Hercules, CA, USA). Primary antibody sources: AMPK (#5831, CST, Danvers, MA, USA), p-AMPK (#2535, CST, Danvers, MA, USA), CD36 (#28109, CST, Danvers, MA, USA), DPP4 (#61408, CST, Danvers, MA, USA), PPARα (#340843, Zenbio, Chengdu, China).

### 4.16. Quantitative RT-PCR

Total RNA was isolated from liver samples using TRIzol reagent (Takara Bio, Inc., Kusatsu, Japan) following the manufacturer’s protocol. Equal amounts of RNA were reverse-transcribed into cDNA using a Takara kit. Gene expression was quantified via RT-qPCR on an Applied Biosystems system (Thermo Fisher Scientific, Waltham, MA, USA) with SYBR Green (Takara Bio, Inc., San Jose, CA, USA). Expression levels were normalized to the internal control *Gapdh*. Primer sequences are listed in Supplementary Table S4. All qPCR procedures (primer design, quantification, and PCR validation) followed previously described methods.

### 4.17. Statistical Analysis

All results are expressed as mean ± SD, unless otherwise noted. Multiple group comparisons were performed using one-way ANOVA followed by Tukey’s post hoc test. Differences between two groups were assessed with Student’s *t*-test. A *p*-value < 0.05 was considered statistically significant. Column charts and box plots were created with GraphPad Prism (v7.0, GraphPad Software, Boston, MA, USA), which was also used for all statistical analyses. The positive staining areas of Nile Red and Oil Red O images were quantified using ImageJ software (v1.53a, National Institutes of Health, Bethesda, MD, USA). Heatmap data were standardized using the Z-score method (z = (x − μ)/σ).

## 5. Conclusions

**Figure 9:**
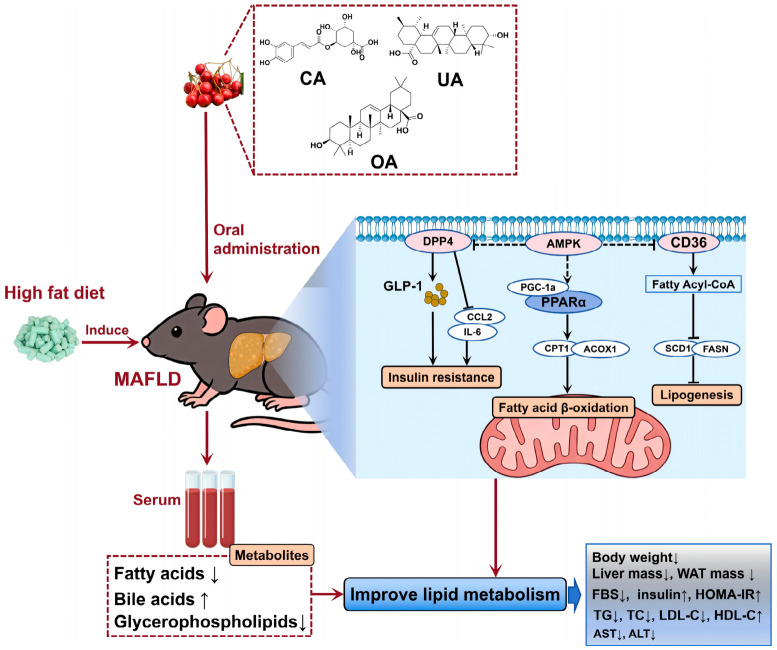
Schematic illustration of the proposed protective mechanism of CFE against HFD-induced MAFLD in mice. Based on the findings of this study, CFE and its candidate active components (chlorogenic acid, ursolic acid, and oleanolic acid) are associated with activation of the AMPK-PPARα pathway and suppression of CD36 and DPP4. These changes may contribute to reduced hepatic lipid accumulation, improved insulin sensitivity, and alleviation of MAFLD. Arrows indicate positive regulation; T-bars indicate inhibition.

In conclusion, this study identifies CA, UA, and OA as candidate key bioactive components in CFE responsible for its lipid-lowering effects. CFE treatment significantly reduced body weight gain, improved glucose metabolism, and alleviated hepatic steatosis and liver injury in HFD-induced MAFLD mice. Multi-omics analysis revealed that CFE rectified disturbances in serum fatty acids, glycerophospholipids, and bile acid metabolites, with enrichment of the AMPK/PPAR signalling pathway. Molecular experiments confirmed that CFE significantly upregulated AMPK and PPARα and downregulated CD36 and DPP4 (Figure 9). Nevertheless, the link between lipid metabolite regulation and the AMPK/PPARα/CD36/DPP4 axis, as well as the potential synergy of CA, UA, and OA, requires further investigation. In summary, this study provides multi-angle insights into the potential active components and mechanisms by which CFE ameliorates hepatic steatosis and lipid disturbances, including the regulation of lipid metabolic pathways and involvement of the AMPK/PPARα/CD36/DPP4 signalling axis. These findings provide a deeper mechanistic understanding of the lipid-lowering effects of CFE and support its further development as a functional food ingredient for metabolic health.

## Data Availability

The original contributions presented in this study are included in the article/Supplementary Material. Further inquiries can be directed to the corresponding author.

## Supplementary Materials

The following supporting information can be downloaded at: https://www.mdpi.com/article/10.3390/molecules31122118/s1, Figure S1. Total ion chromatograms (TIC) of serum metabolomics in positive and negative ion modes; Figure S2. RNA-Seq analysis of gene expression changes in GSEA analysis. (A–D) Main KEGG pathways analyzed using GSEA; (E–H) Main GO pathways analyzed using GSEA; Figure S3. The original bands of protein expression levels of AMPK and p-AMPK (A), PPARα (B), CD36 (C), and DPP4 (D); Figure S4. Enrichment analysis for key targets identified through network pharmacology. (A) KEGG enrichment analysis; (B) Cell composition of Gene Ontology (GO) analysis; (C) Molecular function of GO analysis; (D) Biological pathways of GO analysis; Figure S5 The total ion chromatograms and component analysis results of CF in negative ionization modes; Figure S6. LC-MS spectrum of CF extract (negative ion mode). (A) CFE total ion chromatogram; (B) XIC diagram of extracting chlorogenic acid (CA) from CFE; (C) XIC diagram of extracting maslinic acid (MA) and corosolic acid (CSA) from CFE; (D) XIC diagram of extracting oleanolic acid (OA) and ursolic acid (UA) from CFE; Figure S7. CCK 8 cell viability and dose–response analyses in THLE 2 cells. (A–E) Cytotoxicity assays of ursolic acid (UA), oleanolic acid (OA), maslinic acid (MA), corosolic acid (CSA), and chlorogenic acid (CA) in THLE 2 cells, respectively. (F) Cytotoxicity of free fatty acid (FFA) mixture (palmitic acid:oleic acid = 2:1) in THLE 2 cells. (G,H) Dose response analysis of the lipid lowering effects of five potential active ingredients; Table S1. Related information of identified chemical components in CF extract; Table S2. Contents of five potential active monomers in CFE; Table S3. Molecular docking binding energy (kcal mol^−1^); Table S4. Sequences of quantitative PCR primers. Supplementary Data S1, Supplementary Data S2: detailed information on the DEGs.

## Abbreviations

AMPK	adenosine 5′-monophosphate-activated protein kinase
AST	aspartate aminotransferase
ALT	alanine aminotransferase
CF	crataegi fructus
CA	chlorogenic acid
CSA	corosolic acid
CD36	thrombospondin receptor
DPP4	dipeptidyl peptidase 4
DAMs	differentially accumulated metabolites
DEGs	differentially expressed genes
FFA	free fatty acid
FC	fold change
FBS	fasting blood glucose
FINS	fasting insulin
GESA	gene set enrichment analysis
GLP-1	glucagon-like peptide-1
GTT	glucose tolerance test
HFD	high fat diet
HDL-C	high-density lipoprotein cholesterol
HOMA-IR	homeostatic model assessment-insulin resistance
LDL-C	low-density lipoprotein cholesterol
ITT	insulin tolerance test
MA	maslinic acid
MAFLD	metabolic dysfunction-associated fatty liver disease
OA	oleanolic acid
OPLS-DA	orthogonal partial least squares-discriminant analysis
PCA	principal component analysis
PPARa	peroxisome proliferator-activated receptor alpha
PGC-1α	peroxisome proliferator-activated receptor gamma coactivator 1 alpha
UA	ursolic acid
TC	total cholesterol
TG	triglycerides
WGCNA	weighted gene co-expression network analysis
