# Supplementary material for: Exploring Active Ingredients and Mechanisms of Crataegi fructus Extract in Alleviating MAFLD via the AMPK/PPAR Pathway by Multi-Omics
Source: Molecules. 2026 Jun 16;31(12):2118. doi: 10.3390/molecules31122118 (PMC13306006; doi:10.3390/molecules31122118)
Supplement: Supplementary file 1 [file molecules-31-02118-s001.zip › molecules-4338091-supplementary.pdf]

Supplementary Material

Exploring Active Ingredients and Mechanisms of *Crataegi fructus* Extract in Alleviating MAFLD via the AMPK/PPAR Pathway by Multi-Omics

Xing Yan [1,2], Lulu Zheng [1,2], Yuexiang Xiao [3], Ya Xu [1,2], Qing Xu [1,2], Lihua Zeng [1,2], Siqi Hu [1,2], Deqing Ruan [4,*] and Zhixin Wang [1,2,*]

[1] Jiangxi Province Key Laboratory of Sustainable Utilization of Traditional Chinese Medicine Resources & Jiangxi Province Key Laboratory of Traditional Chinese Medicine Pharmacology, Institute of Traditional Chinese Medicine Health Industry, China Academy of Chinese Medical Sciences, Nanchang 330115, China; yanxing@itcmhi.ac.cn (X.Y.); zhenglulu@itcmhi.ac.cn (L.Z.); xuya@itcmhi.ac.cn (Y.X.); xuqing20000402@163.com (Q.X.); zenglihua@itcmhi.ac.cn (L.Z.); hsqjks@163.com (S.H.)

[2] Jiangxi Health Industry Institute of Traditional Chinese Medicine, Nanchang 330115, China

[3] School of Pharmacy, Jiangxi University of Chinese Medicine, Nanchang 330004, China; xiaoyuexiang888@163.com

[4] Yunnan Key Laboratory of Southern Medicine Utilization, Yunnan University of Chinese Medicine, Kunming 650500, China

* Correspondence: ruandeqing@ynucm.edu.cn (D.R.); wangzhixin@itcmhi.ac.cn (Z.W.); Tel.: +86-0791-83069969 (Z.W.)

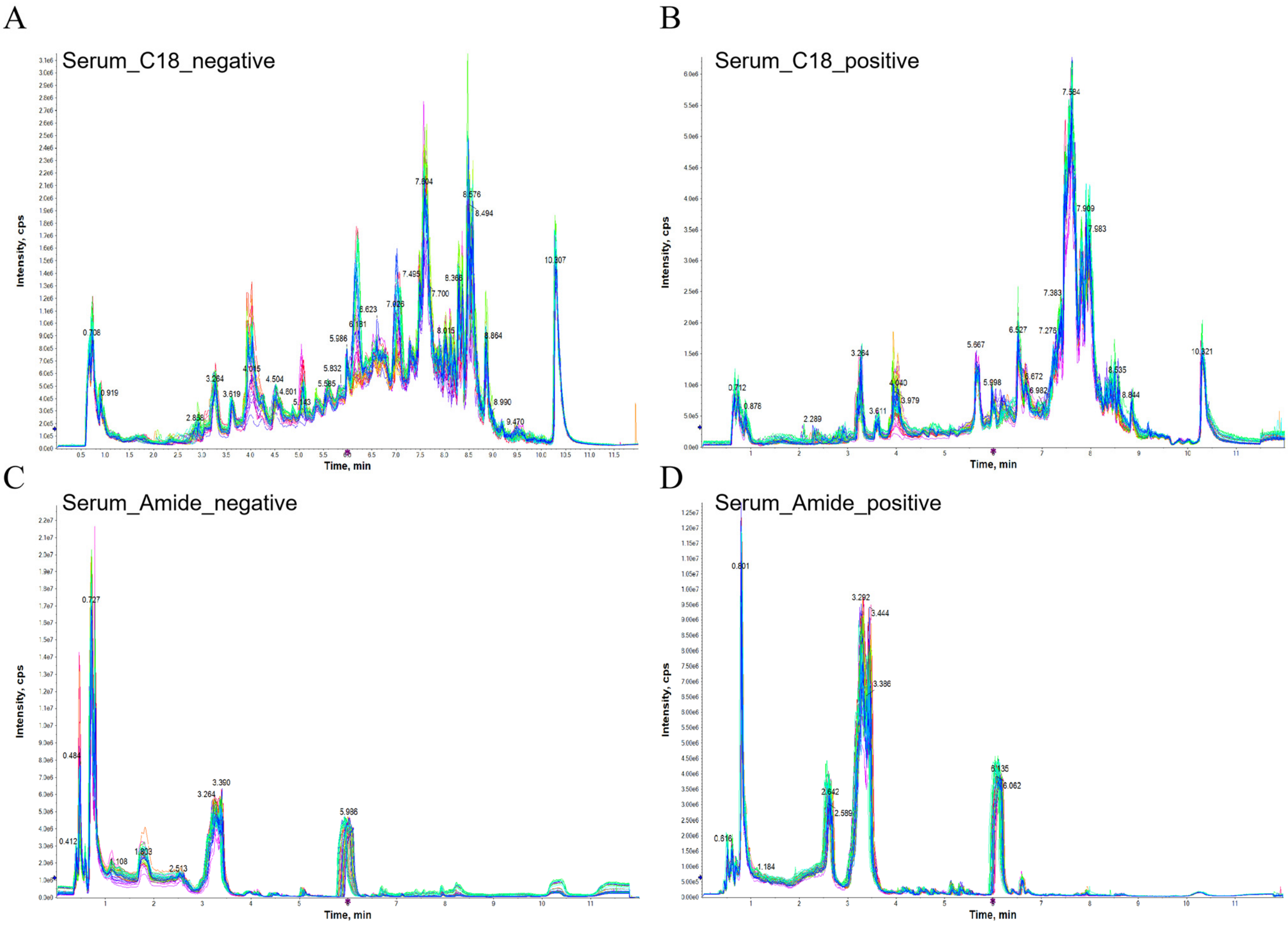

Figure S1. Total ion chromatograms (TIC) of serum metabolomics in positive and negative ion modes.

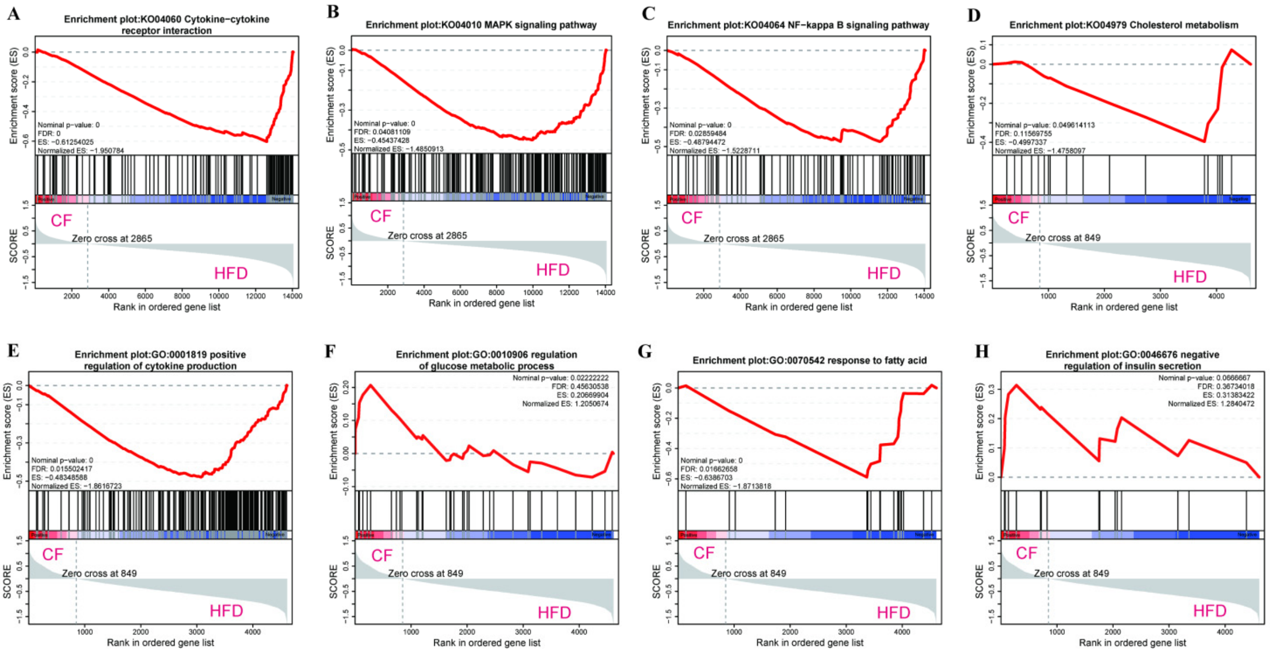

Figure S2. RNA-Seq analysis of gene expression changes in GSEA analysis. (A-D) Main KEGG pathways analyzed using GSEA; (E-H) Main GO pathways analyzed using GSEA.

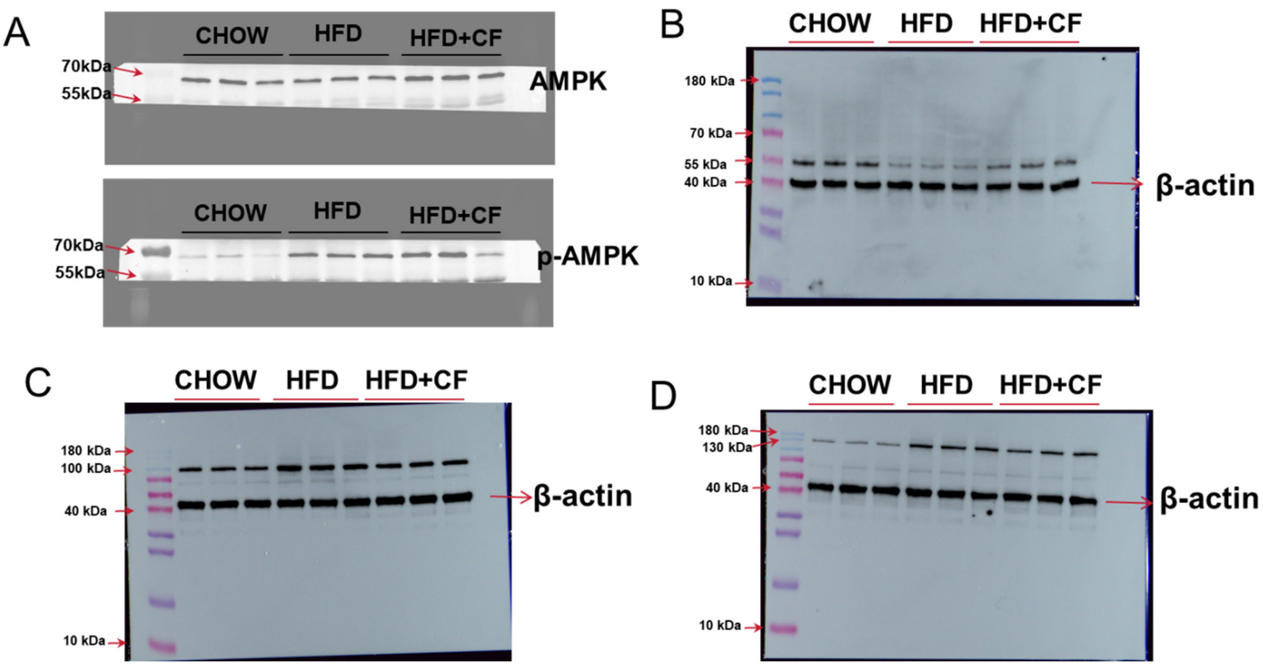

Figure S3. The original bands of protein expression levels of AMPK and p-AMPK (A), PPARα (B), CD36 (C), and DPP4 (D).

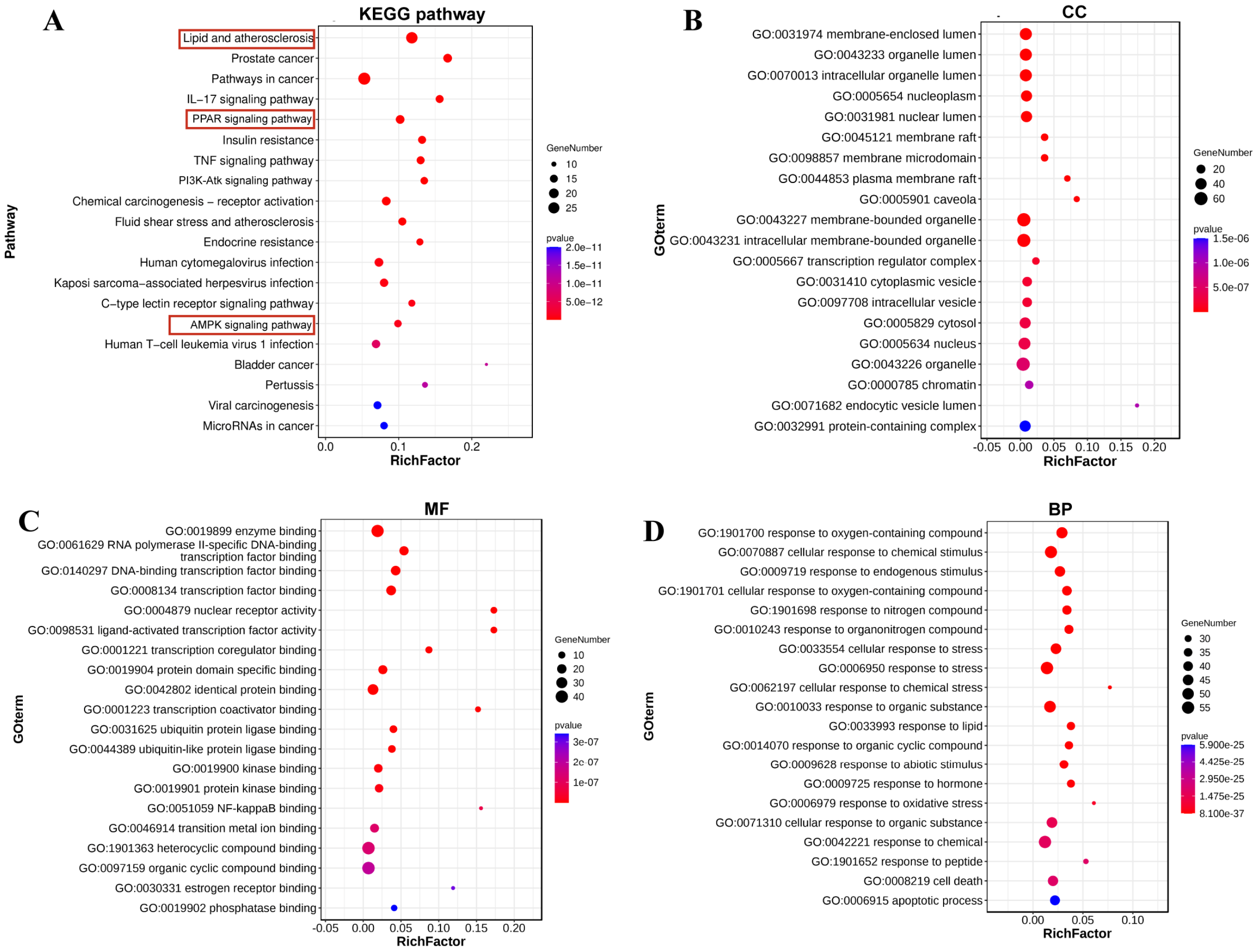

Figure S4. Enrichment analysis for key targets identified through network pharmacology. (A) KEGG enrichment analysis; (B) Cell composition of Gene Ontology (GO) analysis; (C) Molecular function of GO analysis; (D) Biological pathways of GO analysis.

UHPLC/Q-TOF-MS analysis for identification of CF components

Qualitative analysis was performed using an XionLC-X500R QTOF system (AB Sciex, Framingham, USA) with an ACQUITY UPLC HSS T3 column (100×2.1 mm, 1.8 μm) (Waters, Milford, MA, USA) at 40 °C. The mobile phases were 0.02% formic acid in water (A) and methanol:acetonitrile (v/v=1:1) (B), and gradient elution was performed as follows: 5% B (0–2 min), 5–25% B (2–9 min), 25–40% B (9–15 min), 40-55% B (15–19 min), 55–70% B (19–23 min), 70–95% B (23–25 min), and 95–100% B (25–30 min). The flow rate was 0.3 mL/min, with an injection volume of 5 μL. The electrospray ionization (ESI) source was operated in both positive and negative ionization modes, with capillary voltages set at 5500 V for positive ionization and 4500 V for negative ionization. Information-dependent acquisition (IDA) was employed with the following parameters: a declustering potential (DP) of 60 V, a collision energy (CE) of 10 V, an accumulation time of 0.2 s, and a mass range of 100–1500 Da. For MS/MS analysis, dynamic background subtraction was applied, excluding candidate ions for 6 s after two occurrences, with a maximum of 12 candidate ions, and the parameters were as follows: a DP of 60 V, a CE of 20 ± 15 V, an accumulation time of 0.05 s, and a mass range of 50–1500 Da.

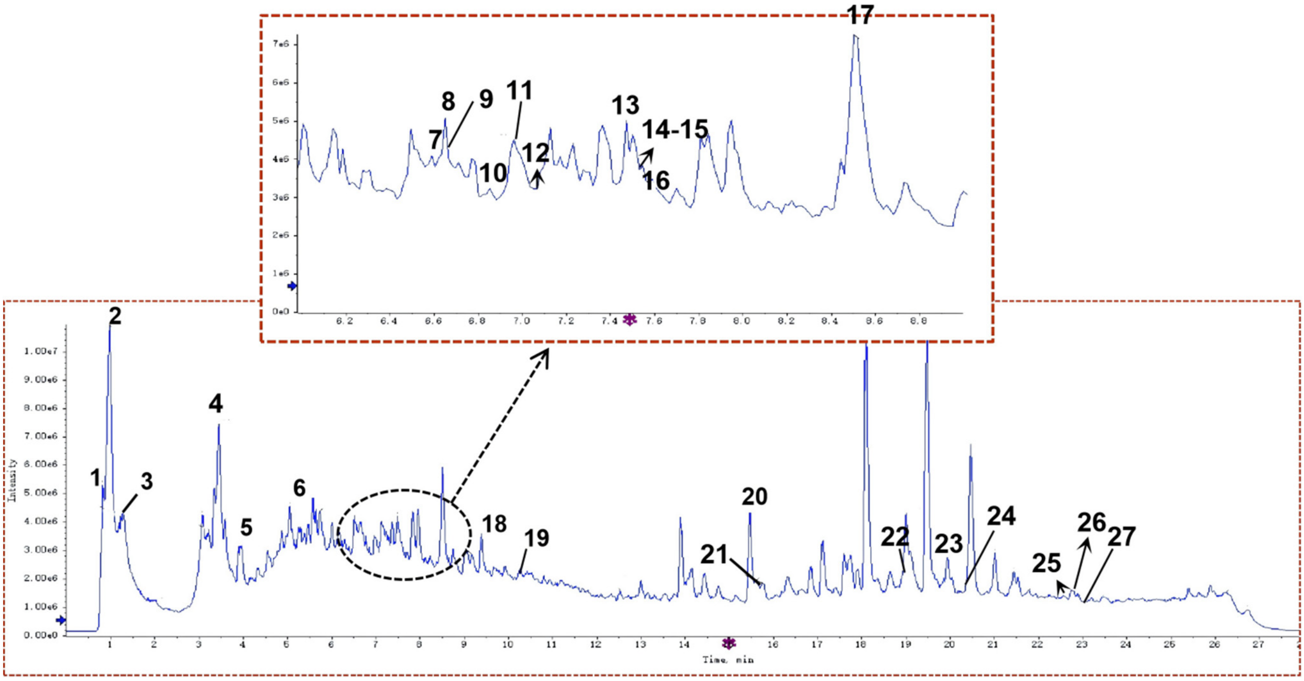

Figure S5 The total ion chromatograms and component analysis results of CFE in negative ionization modes.

Table S1. Related information of identified chemical components in CFE

No.	Chemical compound	Rt (min)	Mass (m/z)	Proposal ions	MS^2 fragment ions (m/z)
1	Malic acid	0.84	133.0142	[M-H]	115.0037, 71.0139
2	Citric acid	0.95	191.1087	[M-H]	111.0088, 87.0087, 67.0195
3	Succinic acid	1.23	117.0194	[M-H]	73.0285
4	Protocatechuic acid	3.78	153.0193	[M-H]	109.0297, 91.0191, 81.0360
5	Chlorogenic acid	3.96	353.0869	[M-H]	191.0546, 707.1852
6	Caffeic acid	3.83	179.0315	[M-H]	135.1009, 59.0145
7	vitexin-4′-rhamnosyl	5.023	577.1555	[M-H]	293.0435, 413.0833, 577.1533
8	kaempferol-3-O-neohesperidoside	6.687	7.0118	[M-H]	593.2091, 289.0693, 441.1174
9	vitexin-4′-O-glucoside	6.600	593.1501	[M-H]	293.0435, 413.0833, 593.1501
10	vitexin-2″-O-rhamnosyl	6.688	577.1555	[M-H]	293.0435, 413.0833, 577.1533
11	(p-hydroxyphenyl) benzoic acid	6.708	267.0882	M-H]	149.0614, 107.0520, 133.0650
12	vitexin	6.81	431.0972	[M-H]	312.0577, 342.0684, 284.0627

13	rutin	6.981	611.1456	[M+H]	302.0273, 303.0353, 273.0247
14	hyperoside	7.11	463.0875	[M-H]	300.0267, 271.0243, 255.0301
15	vitexin-6″-O-acetyl	7.497	473.1061	[M-H]	473.1061, 289.0710, 245.0850
16	vitexin-2″-O-acetyl	7.679	473.1061	[M-H]	473.2178, 289.0707, 245.0868
17	Linarionoside A	7.46	375.1430	[M+H]	147.0438, 179.0698, 161.0598
18	Linarionoside B	7.53	375.1430	[M+H]	147.0438, 179.0698, 161.0598
19	kaempferol	8.458	331.1539	[M+FA-H]	287.1283, 137.0594, 151.0743
20	quercetin	9.49	301.0352	[M-H]	151.0033, 79.5992, 178.9996
21	Gallic acid	10.13	169.124	[M-H]	125.0951, 169.1240
22	astragalin	6.53	449.3039	[M+H]	85.0275, 97.0282, 127.0367
23	Linarionoside C	18.91	537.3020	[M+H]	520.3364, 353.2665, 423.3592
24	Crataegolic acid	19.924	471.3464	[M-H]	471.3480, 507.3, 423.3123
25	Corosolic acid	20.258	471.3464	[M-H]	471.3480, 507.3, 423.3123
26	Oleanolic acid	22.45	455.349	[M-H]	406.9100, 455.3516, 279.2331
27	Ursolic acid	22.83	455.39	[M-H]	406.9100, 455.3516, 279.2331
28	arjungenin	22.83	502.3799	[M-H]	455.3483, 456.3547

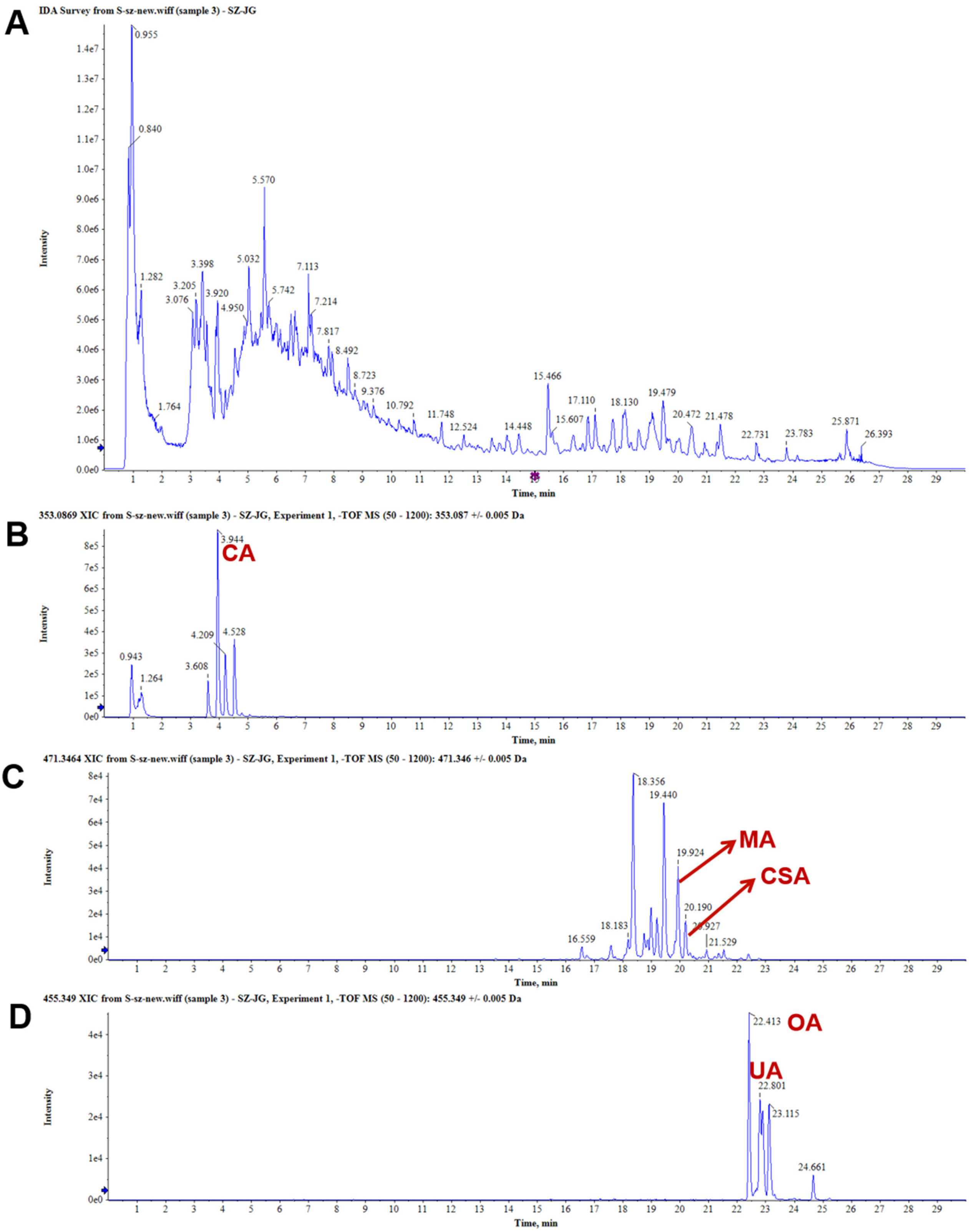

Figure S6. LC-MS spectrum of CFE (negative ion mode). (A) CFE total ion chromatogram; (B) XIC diagram of extracting chlorogenic acid (CA) from CFE; (C) XIC diagram of extracting maslinic acid (MA) and corosolic acid (CSA) from CFE; (D) XIC diagram of extracting oleanolic acid (OA) and ursolic acid (UA) from CFE.

Table S2. Contents of five potential active ingredients in CFE.

Chemical compound	ug/mg	RSD % (n=3)
UA	10.81	0.27
OA	9.02	1.26
MA	0.27	0.95
CSA	0.59	2.63
CA	18.17	1.44

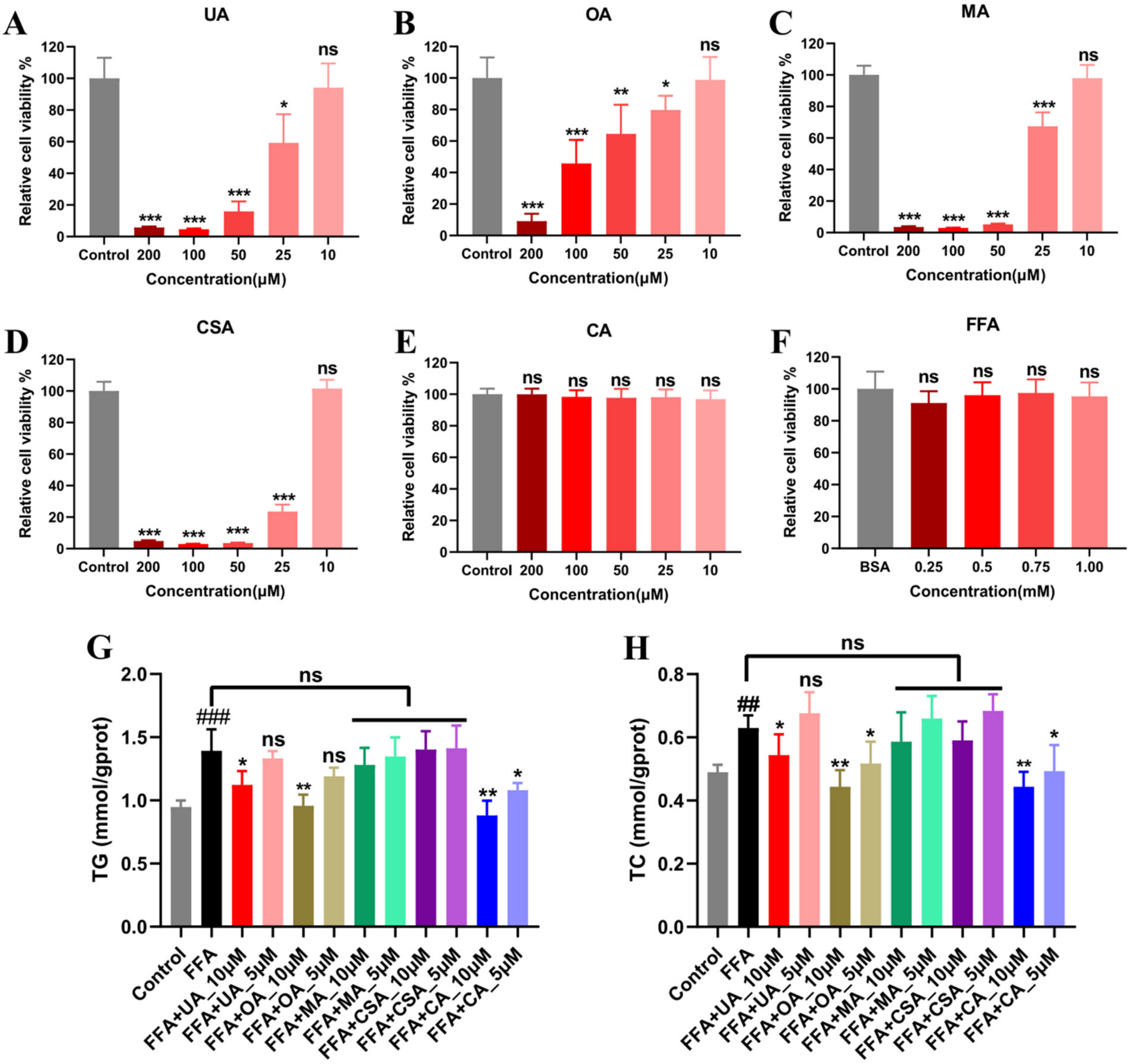

Figure S7. CCK-8 cell viability and dose–response analyses in THLE-2 cells. (A–E) Cytotoxicity assays of ursolic acid (UA), oleanolic acid (OA), maslinic acid (MA), corosolic acid (CSA), and chlorogenic acid (CA) in THLE-2 cells, respectively. (F) Cytotoxicity of free fatty acid (FFA) mixture (palmitic acid : oleic acid = 2 : 1) in THLE-2 cells. (G, H) Dose-response analysis of the lipid-lowering effects of five potential active ingredients.

Table S3. Molecular docking binding energy (kcal mol^{-1})

Index	PDB ID	Chlorogenic acid	Corosolic acid	Ursolic acid	Maslinic acid	Oleanolic acid	Kaempferol	Quercetin
CD36	5LGD	-8.1	-1.5	-1.8	-1.6	-2.9	-7.5	-7.5
DPP4	1RWQ	-7.3	-8.3	-8.2	-8.6	-8.8	-2.1	-3.3
PPARα	2p54	-7.5	-5.1	-5.9	-5.4	-5.4	-4.8	-4.7

Table S4. Sequences of quantitative PCR primers

**Gene**	**Forward Primer (5′→3′)**	**Reverse Primer (5′→3′)**	**GeneBank Accession NO.**
Srebp-1c	CTTTGGCCTCGCTTTTCGG	TGGGTCCAATTAGAGCCATCTC	NM_011480
Acs	GCTGCCGACGGGATCAG	TCCAGACACATTGAGCATGTCAT	NM_007981
Scd-1	TTCTTGCGATACACTCTGGTGC	CGGGATTGAATGTTCTTGTCGT	NM_009127
Cd36	GTCAACATATTGGTCAAGCCGC	CCACTCCAATCCCAAGTAAGGC	NM_007643
Mogat1	TTGACCCATGGTGCCAGTTT	GTGGCAAGGCTACTCCCATT	NM_026713
Fatp5	TTCGAAAGAACCAACCCTTCCT	GCGTCGTACATTCGCAACAA	NM_009512
Fasn	AAGCGGTCTGGAAAGCTGAA	AGGCTGGGTTGATACCTCCA	NM_007988
Cpt1a	GCACACCAGGCAGTAGCTTT	CAGGAGTTGATTCCAGACAGGT	NM_013495
Acat1	CCCCATTGATTTTCCACTTG	AGCACAACCACACTGAATGC	NM_144784
Acox1	CACGGCTATTCTCACAGCAG	CAGGCTGTTAATGTCCACCA	NM_015729
Pparα	AACATCGAGTGTCGAATATGTGG	CCGAATAGTTCGCCGAAAGAA	NM_011144
Gapdh	TGTCCACCTTCCAGCAGATGT	AGCTCAGTAACAGTCCGCCTAGA	NM_007392
